# Janus-faced Sestrin2 controls ROS and mTOR signalling through two separate functional domains

**DOI:** 10.1038/ncomms10025

**Published:** 2015-11-27

**Authors:** Hanseong Kim, Sojin An, Seung-Hyun Ro, Filipa Teixeira, Gyeong Jin Park, Cheal Kim, Chun-Seok Cho, Jeong-Sig Kim, Ursula Jakob, Jun Hee Lee, Uhn-Soo Cho

**Affiliations:** 1 Department of Biological Chemistry, University of Michigan, Ann Arbor, Michigan 48109, USA; 2 Department of Molecular & Integrative Physiology, University of Michigan, Ann Arbor, Michigan 48109, USA; 3 Department of Molecular, Cellular and Developmental Biology, University of Michigan, Ann Arbor, Michigan 48109, USA; 4 Infection and Immunity Unit, Instituto de Investigação e Inovação em Saúde, Universidade do Porto, 4200 Porto, Portugal; 5 IBMC—Instituto de Biologia Molecular e Celular, Universidade do Porto, 4150-180 Porto, Portugal; 6 ICBAS—Instituto de Ciências Biomédicas Abel Salazar, Universidade do Porto, 4050-313 Porto, Portugal; 7 Department of Fine Chemistry, Seoul National University of Science and Technology, Seoul 139-743, Korea; 8 Department of Obsterics and Gynecology, Soonchunhyang University Seoul Hospital, Seoul 140-743, Korea

## Abstract

Sestrins are stress-inducible metabolic regulators with two seemingly unrelated but physiologically important functions: reduction of reactive oxygen species (ROS) and inhibition of the mechanistic target of rapamycin complex 1 (mTORC1). How Sestrins fulfil this dual role has remained elusive so far. Here we report the crystal structure of human Sestrin2 (hSesn2), and show that hSesn2 is twofold pseudo-symmetric with two globular subdomains, which are structurally similar but functionally distinct from each other. While the N-terminal domain (Sesn-A) reduces alkylhydroperoxide radicals through its helix–turn–helix oxidoreductase motif, the C-terminal domain (Sesn-C) modified this motif to accommodate physical interaction with GATOR2 and subsequent inhibition of mTORC1. These findings clarify the molecular mechanism of how Sestrins can attenuate degenerative processes such as aging and diabetes by acting as a simultaneous inhibitor of ROS accumulation and mTORC1 activation.

## Abstract (web summary)

Sestrins are conserved stress-inducible metabolic regulators implicated in the prevention of agerelated diseases. Here, Kim *et al.* report the crystal structure of human Sestrin2 and propose a molecular mechanism for how Sestrin2 functions to prevent ROS accumulation and inhibit mTORC1 activity.

## Graphical abstract

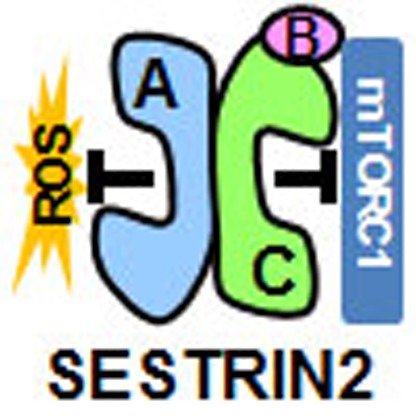

Sestrins are a family of stress-inducible metabolic regulators^1^ that are conserved throughout the metazoan species. Cell-based studies showed that Sestrins have an antioxidant function that suppresses reactive oxygen species (ROS)^2^. In addition to its antioxidant activity, Sestrins activate AMP-activated protein kinase (AMPK) and subsequently inhibit mechanistic target of rapamycin (mTOR) complex 1 (mTORC1)^3^. Genetic studies of *Drosophila* Sestrin (dSesn) revealed that dSesn also functions as a critical negative feedback regulator of dTORC1 (ref. 4). Depletion of dSesn downregulates AMPK and upregulates dTORC1, which together lead to the accelerated development of several age-related and obesity-induced pathologies, such as lipid accumulation, mitochondrial dysfunction, protein aggregate formation, cardiac arrhythmia and muscle degeneration^4^. These pathologies are very reminiscent of age-associated human diseases, which are promoted by obesity. Importantly, most of the observed pathologies were suppressed by administration of AMPK activators, mTORC1 inhibitors or antioxidants^4^, indicating that the mTORC1- and ROS-controlling functions of Sestrin are indeed important for its physiological functions. Similar age-associated metabolic defects were also observed in cSesn-mutated *Caenorhabditis elegans*^5^, suggesting that the physiological roles of Sestrin-family proteins are evolutionarily conserved.

Indeed, Sestrins in mammals also play an important metabolism-regulating role. Recent studies indicate that mouse Sestrins are important for attenuating obesity-associated metabolic liver pathologies such as insulin resistance and steatohepatitis through oxidative stress suppression^6^ or AMPK activation, mTORC1 inhibition and subsequent mTORC2 potentiation^7^,^8^,^9^. Also in other tissues such as lungs^10^,^11^, kidneys^12^, neurons^13^,^14^,^15^, macrophages^16^, as well as embryonic fibroblasts^17^, mammalian Sestrins are important for proper regulation of ROS or mTORC1 signalling.

Although it is clear that Sestrins are critical for ROS- and mTOR-associated metabolic homeostasis, we still do not have a clear biochemical understanding of how a single protein can perform such a diverse set of physiological roles, crucially important for metabolic homeostasis and aging prevention. This is mostly due to the complete lack of structural information about Sestrins. Here we report the crystal structure of human Sestrin2 (hSesn2) for the first time. The structure of hSesn2 displays an interesting internal symmetry with two homologous subdomains (Sesn-A and Sesn-C), which have a similar structure but distinct functions. Sesn-A functions as an alkylhydroperoxide reductase, while Sesn-C performs an mTORC1-inhibiting role. Through these two independent domains, Sestrin can single-handedly suppress both ROS and mTORC1, which are well-established promoters of aging and age-associated pathologies^18^. Therefore, our discovery provides an explanation for how a single protein can play such a versatile anti-aging role, especially considering that excessive ROS accumulation and chronic mTORC1 activation are well-known facilitators of aging and age-associated diseases.

## Results

### Structural determination of full-length hSesn2 protein

**Figure 1:**
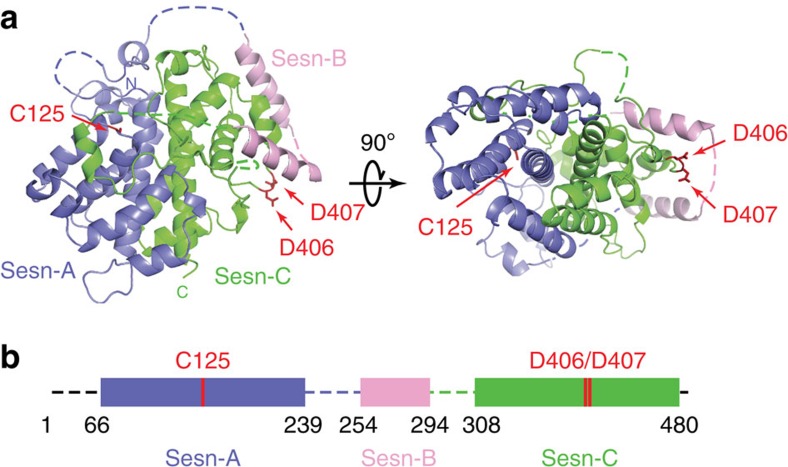
Crystal structure of full-length hSesn2. (**a**) Ribbon diagram of full-length hSesn2. Sesn-A, Sesn-B and Sesn-C domains are in slate, pink and green, respectively. hSesn2 is composed of two globin-like α-helix-only domains (Sesn-A and Sesn-C) connected by a helix–loop–helix domain (Sesn-B) with a total of 23 helices. The overall structure is well-defined except for residues 1–65, 221–224, 240–255, 272–279, 295–307, 329–332 and 479–480. Key residues (C125, D406 and D407) in each of the globular domains are displayed in a stick model, indicated by red arrows. (**b**) Schematic diagram of domain organization of hSesn2. Illustrations of the protein structure used in all figures were generated with either PYMOL (Delano Scientific, LLC) or Chimera (UCSF chimera). The relative locations of C125, D406 and D407 are marked in red.

To gain insights into the biochemical and structural properties of Sestrins, we determined the crystal structure of full-length hSesn2 at 3.5-Å resolution (Fig. 1a and Table 1). The electron density map of hSesn2 was calculated using the single-wavelength anomalous diffraction (SAD) method with selenomethionine (SeMet)-substituted proteins. Despite its low-resolution diffraction, we were able to obtain a high-quality electron density map by taking advantage of non-crystallographic symmetry averaging (five copies of hSesn2 per asymmetric unit (ASU)) and the high solvent content (68%) (Supplementary Fig. 1). Furthermore, the helix-dominant structure and appropriately located selenium positions (total 12 sites per monomer) allowed us to precisely place the sequence in the electron density map. The final refined model of hSesn2 shows an *R*/*R*_free_ of 24.3/26.9.

### Overall structure of hSesn2 protein

**Figure 2:**
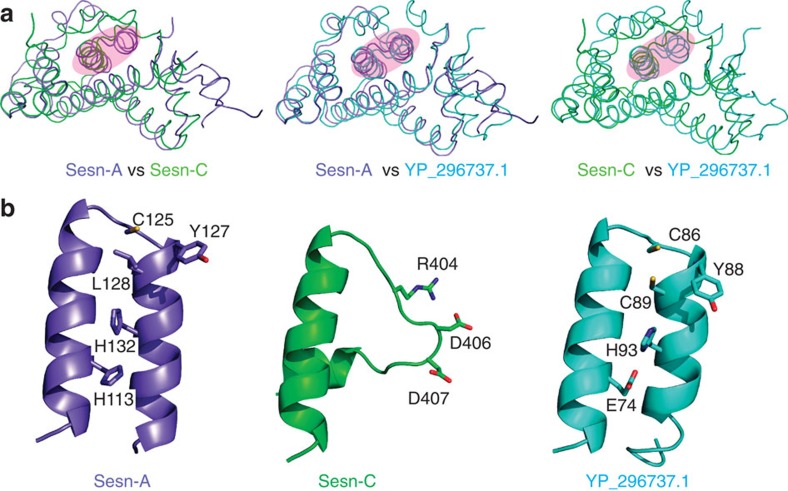
hSesn2 subdomains (Sesn-A/Sesn-C) have a structural similarity to YP_296737.1. (**a**) Structural overlay between Sesn-A (slate), Sesn-C (green) and monomeric *R. eutropha* YP_296737.1 (cyan). The overall architectures of Sesn-A, Sesn-C and YP_296737.1 are structurally similar to each other with r.m.s. differences of 1.95 Å (Sesn-A versus Sesn-C, total 110 residues compared), 1.94 Å (Sesn-A versus YP_296737.1, 139 residues) and 2.32 Å (Sesn-C versus YP_296737.1, 104 residues). From this study, we identified two functionally active sites in each of Sesn-A and Sesn-C domains, which are highlighted in pink. (**b**) Structure comparison of the highlighted regions in **a**, which corresponds to the helix–turn–helix oxidoreductase motif of YP_296737.1. Only one cysteine is preserved in Sesn-A (Cys125), and none are found in Sesn-C.

The crystal structure revealed that hSesn2 belongs to the family of globin-like α-helix-fold proteins, consisting of 23 helices and no β-sheets (Fig. 1a and Supplementary Fig. 2a,c). The structure of full-length hSesn2 indicates that the protein contains three domains separated by two unstructured flexible linker regions (Fig. 1b). These three domains correspond well to the Sesn-A, Sesn-B and Sesn-C domains, which have been previously identified through a phylogenetic analysis^19^. Both Sesn-A and Sesn-C domains of hSesn2 form a globular shape, which is noticeably similar to each other (Figs 1a and 2a). Indeed, the Cα r.m.s. difference between Sesn-A and Sesn-C turned out to be only 1.95 Å (total 110 residues compared). Although Sesn-B was originally predicted to have a coiled coil motif (Supplementary Fig. 3), it forms a helix–loop–helix structure and lies on the surface of Sesn-C (Fig. 1a). hSesn2 appears to be a monomer, both in its crystal structure (Supplementary Fig. 1b) as well as in its analysis using size-exclusion chromatography combined with multi-angle light scattering (Supplementary Fig. 4).

### Structural similarity between Sesn-A and oxidoreductases

To infer the function of hSesn2 based on its three-dimensional structure, we searched for proteins structurally related to hSesn2 using a distance-matrix alignment programme (Dali server)^20^. The top ranked result revealed that both Sesn-A and Sesn-C possess a high degree of structural similarity with an uncharacterized protein YP_296737.1 (PDB ID: 2PRR) in *Ralstonia eutropha* JMP134 (Fig. 2a and Supplementary Fig. 5a). Interestingly, the Sesn-A and Sesn-C domains in the full-length hSesn2 protein overlap with the dimer structure of YP_296737.1 (Supplementary Fig. 6), suggesting that the monomer of YP_296737.1 has been duplicated in hSesn2, and divergently evolved into two domains in a single polypeptide.

YP_296737.1 was predicted as a putative alkylhydroperoxidase^21^. Despite barely conserved primary sequences (Supplementary Fig. 5b), we noted that 109–139 amino acids of the Sesn-A domain show a very distant sequence homology to YP_296737.1 as well as to AhpD, a well-characterized alkylhydroperoxidase in *Mycobacterium tuberculosis*^21^,^22^,^23^, as formerly reported^2^. The homology region corresponds to the helix–turn–helix motif of AhpD, a signature motif found in the family of AhpD-like oxidoreductases^21^,^22^,^23^. The relative position of the motif within the primary sequence is similar between hSesn2 and YP_296737.1 but different in AhpD (Supplementary Fig. 5a). Interestingly, whereas both YP_296737.1 and AhpD are characterized by a catalytic cysteine dyad (Cys86 and Cys89 in YP_296737.1), the Sesn-A domain contains only the first cysteine (Cys125 in hSesn2) with the second cysteine being substituted for a leucine (Leu128 in hSesn2) (Fig. 2b). Nevertheless, other catalytically important residues proposed to mediate the proton relay system in AhpD^23^, are well-conserved in the Sesn-A domain (Tyr127, His132 and His113) as well as in YP_296737.1 (Tyr88, His93 and Glu74) (Fig. 2b and Supplementary Fig. 5b).

### Sesn-A functions as an alkylhydroperoxide reductase

**Figure 3:**
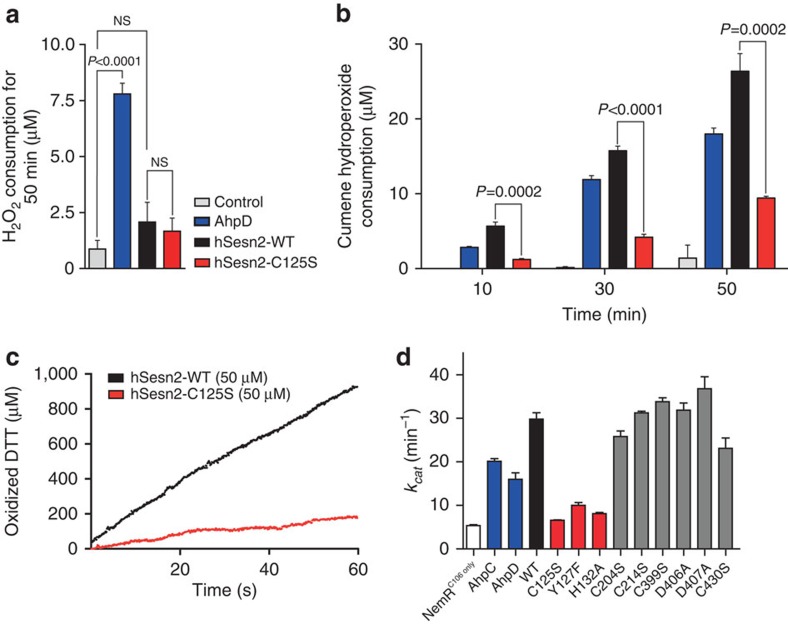
hSesn2 is an alkylhydroperoxidase using a single catalytic cysteine in Sesn-A. (**a**) hSesn2 does not show significant peroxidase activity against H_2_O_2_. Ferrous oxidation–xylenol orange (FOX) assay was used to quantify the amount of remaining H_2_O_2_ after reaction with DTT catalysed by *M. tuberculosis* AhpD, hSesn2-WT or hSesn2-C125S. Total H_2_O_2_ consumption amounts for the initial 50 min are measured and presented as a bar graph (*n*=3, mean±s.e.m.). *P* values were calculated using the Student's *t*-test. NS, non-significant (*P*>0.05). (**b**) From the FOX assay, hSesn2 shows significant peroxidase activity against cumene hydroperoxide, which is dependent on Cys125. The cumene hydroperoxide consumption for the initial 10, 30, 50 min are presented as a bar graph (*n*=3, mean±s.e.m.). *P* values were calculated using the Student's *t*-test. (**c**) Dithiothreitol (DTT)-dependent alkylhydroperoxidase activity of hSesn2 (WT and C125S mutant) towards cumene hydroperoxide was measured at 310 nm. (**d**) The *k*_cat_ of hSesn2-WT, NemR^C106 only^ (negative control, white), *M. tuberculosis* AhpC/AhpD (blue), and hSesn2-WT and hSesn2-mutants was presented as a bar graph (*n*=3, mean±s.e.m.).

To investigate whether hSesn2 is indeed a functional antioxidant enzyme *in vitro*, we tested its ROS-detoxifying activities using different substrates. Even though AhpD is able to reduce hydrogen peroxide^24^, hSesn2 did not exhibit any detectable reducing activities towards hydrogen peroxide (Fig. 3a). However, cumene hydroperoxide, an alkylhydroperoxide with a bulky hydrophobic group^24^, was efficiently reduced by hSesn2, as shown by two independent biochemical assays involving quantification of reduced alkylhydroperoxides (Fig. 3b) or oxidized dithiothreitol (Fig. 3c). These results suggest that hSesn2 is an active alkylhydroperoxide-detoxifying enzyme. Mutation of Cys125 (C125S)—the only catalytic cysteine identified in the putative active site of hSesn2, His132 (H132A)—the residue critical for the conserved proton relay system, or Tyr127 (Y127F)—the residue potentially involved in the catalytic process, reduced this redox activity down to the level of a redox-active yet peroxidase-inactive control protein (that is, NemR^C106 only^)^25^ (Fig. 3d). The difference between the *k*_cat_ values of hSesn2-C125S and NemR^C106 only^ is only ∼5% of the difference between hSesn2-WT and NemR^C106 only^, demonstrating that Cys125 alone can account for ∼95% of alkylhydroperoxidase activity of hSesn2 over the negative control. However, mutations of other surface-exposed cysteines did not abolish the alkylhydroperoxidase activity of hSesn2 (Fig. 3d). These results suggest that the helix–turn–helix motif of the Sesn-A domain indeed constitutes an active catalytic site as an oxidoreductase. It is worth noting that Cys125 is surrounded by hydrophobic molecular surfaces (Supplementary Fig. 7), which may direct hSesn2's peroxidase activity specifically towards hydrophobic alkylhydroperoxides, such as cumene hydroperoxide, and reduce the affinity for hydrogen peroxide. Intriguingly, the *k*_cat_ value of hSesn2-WT (29.82±2.5 min^−1^) is considerably higher than that of *M. tuberculosis* AhpC (20.13±1.03 min^−1^) and AhpD (16.01±2.54 min^−1^), suggesting that hSesn2 is a more effective alkylhydroperoxidase than these bacterial enzymes.

### hSesn2 uses cysteine sulfenic acid as a reaction intermediate

**Figure 4:**
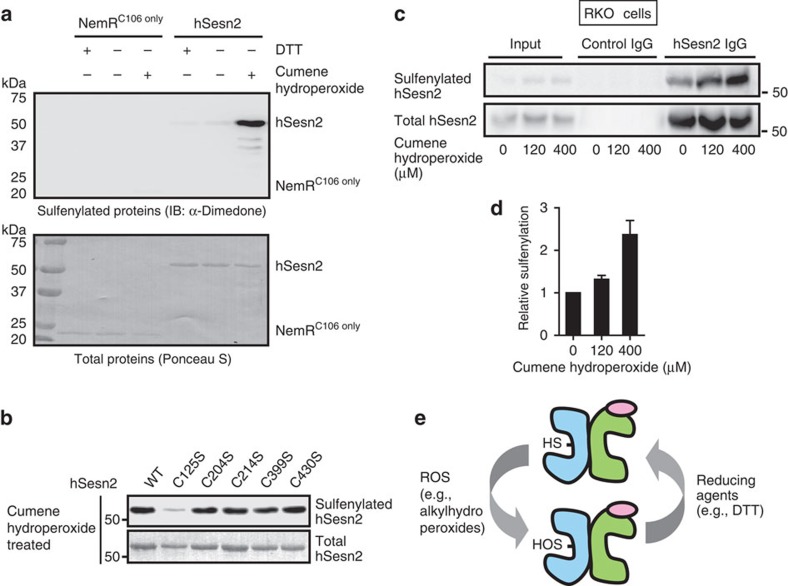
***In vitro*** **and** ***in vivo*** **sulfenylation of Cys125 in hSesn2 by cumene hydroperoxides.** (**a**) Purified NemR^C106 only^ and hSesn2-WT were incubated with 1 mM DTT or 120 μM cumene hydroperoxides and then treated with dimedone, which specifically labels cysteine sulfenic acids, and analysed through anti-dimedone immunoblotting (IB). Ponceau S staining was used to visualize the total levels of hSesn2 proteins. (**b**) Purified hSesn2 proteins of indicated Cys-to-Ser mutations were incubated with 120 μM cumene hydroperoxides. Protein sulfenylation was examined as described in **a**. (**c**) RKO cells were treated with indicated concentrations of cumene hydroperoxide for 20 min. Endogenous hSesn2 was immunopurified using hSesn2 antibodies or pre-immune immunoglobulin (IgG) and analysed by immunoblotting with a non-reducing SDS–PAGE gel. (**d**) Relative protein sulfenylation in **c** was presented as a bar graph (right panel; *n*=4, mean±s.e.m.). (**e**) Schematic diagram of the proposed reaction mechanism underlying hSesn2's peroxidase activity. Cys125 (Cys-SH) is oxidized by hydrophobic alkylhydroperoxides such as cumene hydroperoxide. The resulting sulfenic acid (Cys-SOH) is reduced directly by DTT or other unknown physiological reducing agents. Molecular weight markers are indicated in kDa.

In *M. tuberculosis* AhpD, the reaction of the active site cysteine with hydroperoxides leads to the formation of a highly unstable sulfenic acid, which rapidly interacts with the nearby cysteine residue to form a stable disulfide bond^22^,^23^. Since Cys125 in hSesn2 does not contain another cysteine residue in close vicinity, we predicted that a stable sulfenic acid would be formed as a reaction intermediate. Indeed, we detected significant cysteine sulfenylation in hSesn2-WT after treatment with cumene hydroperoxide, but not in a negative control protein NemR^C106 only^, known to form a sulfenamide switch instead^25^ (Fig. 4a). The C125S mutation, but not the mutation of other cysteines in hSesn2, abolished sulfenic acid formation, confirming that Cys125 is the main catalytic residue that is oxidized during reduction of alkylhydroperoxides (Fig. 4b). Analysis of endogenous hSesn2 immunopurified from hydroperoxide-treated RKO cells further demonstrated that hSesn2 undergoes substantial sulfenylation during oxidative stress *in vivo* (Fig. 4c,d). Collectively, these results show that hSesn2 reduces alkylhydroperoxide radicals using single catalytic cysteine (Cys125), which is reversibly sulfenylated during its catalytic cycle (Fig. 4e).

### Helix–turn–helix motif is not conserved in Sesn-C

Sesn-C also displays structural resemblance to YP_296737.1/Sesn-A (Fig. 2a). However, a major difference was detected at the helix–turn–helix motif (Fig. 2b), which is the signature motif in the YP_296737.1/Sesn-A/AhpD structure (Supplementary Fig. 5a). None of the catalytic residues involved in AhpD oxidoreductase activity^23^ are present in the corresponding region of Sesn-C (Fig. 2b). Furthermore, the helix–turn–helix motif is replaced by a helix–loop structure in Sesn-C, strongly suggesting that Sesn-C may have lost its antioxidant function during evolution, even though the overall architecture is still maintained. The sequence of Sesn-C is, however, very strictly conserved across the Sestrin family (Supplementary Fig. 2a,b), suggesting that, during evolution, Sesn-C may have acquired another important functional role that is independent of the antioxidant function.

### Sesn-C is responsible for mTORC1 inhibition

**Figure 5:**
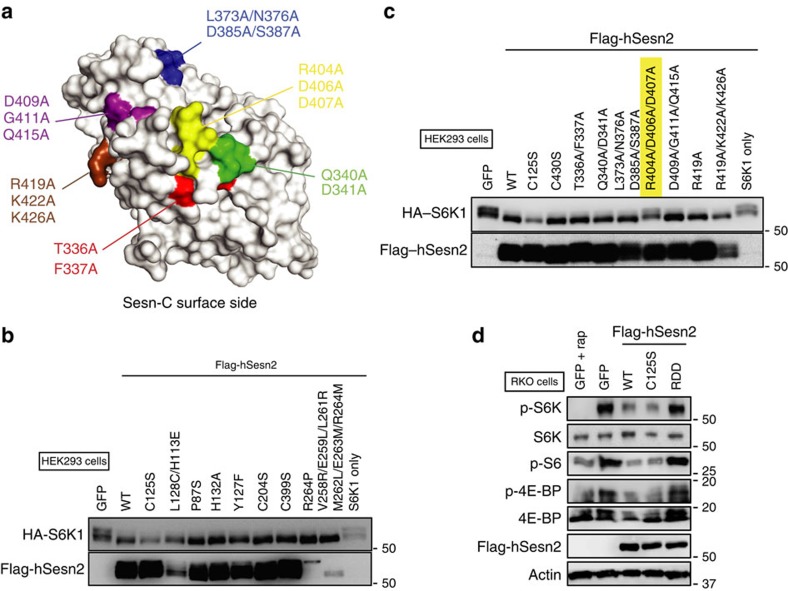
Arg404/Asp406/Asp407 residues in Sesn-C constitute a functional site for mTORC1 regulation. (**a**) The surface of Sesn-C was subdivided into five different areas (highlighted in blue, purple, yellow, green and red) that contain the most highly conserved surface residues. In addition, the residues corresponding to the formerly described putative GDI motif (Arg419, Lys422 and Lys426)^17^ are highlighted in brown. These residues were mutated into alanines as described in Supplementary Table S1. (**b**,**c**) The R404A/D406A/D407A mutation (highlighted in yellow), but none of the other mutations, abolished the mTORC1-inhibiting activity of hSesn2. mTORC1-dependent phosphorylation of S6K was monitored by electromobility retardation of HA–S6K (shifted bands)^26^. HEK293 cells were transfected with plasmid constructs expressing HA-tagged S6K1 and Flag-tagged hSesn2 of the indicated mutations. GFP or an empty vector (S6K1 only) was used as negative controls. After 48 h of transfection, cell lysates were analysed by immunoblotting of the indicated proteins. (**d**) RKO cells were infected with lentiviral constructs expressing GFP or Flag-tagged hSesn2 of indicated mutations. After 48 h of infection, cell lysates were analysed by immunoblotting of the indicated proteins. Rapamycin (rap, 100 nM for 24 h) was used as a positive control for mTORC1 inhibition. Molecular weight markers are indicated in kDa.

**Figure 6:**
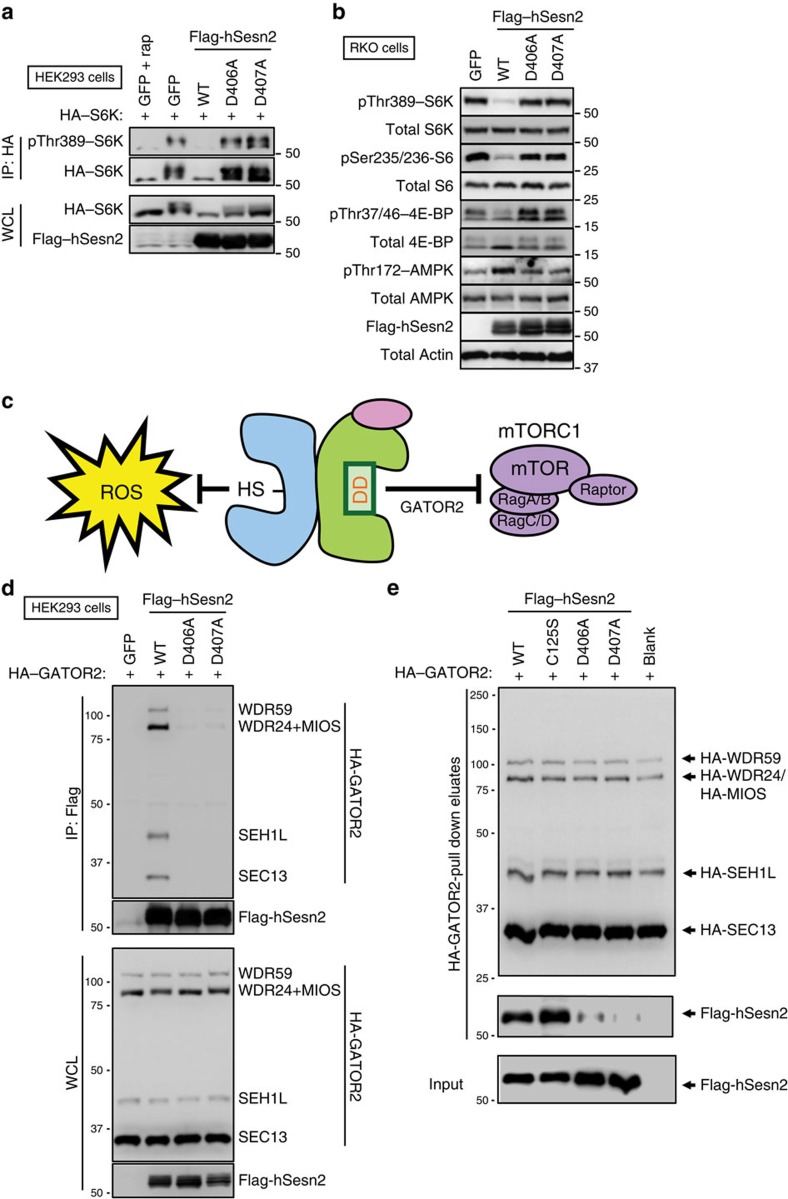
The DD motif in the Sesn-C domain of hSesn2 is responsible for direct binding to the GATOR2 complex. (**a**) HEK293 cells were transfected with plasmid constructs expressing HA-tagged S6K1 and Flag-tagged hSesn2 of indicated mutations or GFP. After 48 h of transfection, HA-immunopurified protein complex (IP) and whole cell lysates (WCL) were analysed by immunoblotting of the indicated proteins. Rapamycin (rap, 100 nM for 24 h) was used as a positive control for mTORC1 inhibition. (**b**) RKO cells were infected with lentiviral constructs expressing GFP or Flag-tagged hSesn2 of indicated mutations. After 48 h of infection, cell lysates were analysed by immunoblotting of the indicated proteins. (**c**) Schematic representation of the molecular functions of hSesn2. hSesn2 is a Janus-faced molecule with two active sites in separate domains. The first site (the helix–turn–helix motif with redox-active cysteine (SH)) functions as an oxidoreductase for alkylhydroperoxide radicals, which damage critical biomolecules such as DNA. The second site (the DD motif) inhibits mTORC1 by binding to GATOR2, a recently discovered mTORC1 regulator. Inhibition of either ROS or mTORC1 can attenuate aging, and Sestrins do both. (**d**) Flag-tagged hSesn2 of indicated mutations was co-transfected with HA-tagged GATOR2 components (WDR59, WDR24, MIOS, SEH1L and SEC13) as indicated. Input (WCL) and Flag-immunopurified protein complex (IP) were analysed by immunoblotting. (**e**) WT, C125S, D406A and D407A mutants of Flag-tagged hSesn2 were purified from transiently transfected HEK293 cells. These proteins were incubated *in vitro* with HA–GATOR2 protein complex bound to anti-HA agarose beads. HA–GATOR2 complexes were then eluted from the beads. The pull-down eluates as well as inputs were analysed by immunoblotting of indicated proteins. Molecular weight markers are indicated in kDa.

To understand the regulatory function of hSesn2 towards mTORC1 inactivation, we introduced a series of structure-guided mutations in hSesn2 (Supplementary Table 1), including mutations in all the evolutionarily conserved surface residues of Sesn-C (Fig. 5a). To test how these mutations affect the mTORC1-suppressing activity of hSesn2, we monitored mTORC1-dependent phosphorylation of p70 S6 kinase (S6K) through a phosphorylation-induced gel shift assay^26^. All the hSesn2 constructs except the R404A/D406A/D407A (hereafter, RDD) mutant maintained their strong suppression of mTORC1, as shown by inhibition of S6K shifts (Fig. 5b,c). The RDD mutation impaired mTORC1-inhibiting activity of hSesn2 in HEK293 (Fig. 5c) and RKO cells (Fig. 5d), as monitored by phosphorylation of mTORC1 downstream proteins such as S6K, S6 and 4E-binding protein (4E-BP). Further analysis of individual mutations within RDD confirmed that both D406A and D407A mutations, but not R404A, abolished the mTORC1-suppressing activity of hSesn2 (Fig. 6a,b and Supplementary Fig. 8a), suggesting that Asp406 and Asp407 (hereafter, the DD motif) play the key role in mTORC1 inhibition. Mutations in the DD motif also prevented hSesn2 from inducing AMPK activation (Fig. 6b), which partially contributes to the mTORC1 suppression^3^,^27^. Interestingly, these two residues are located in the putative active site of Sesn-C (the helix–loop motif, Fig. 2b), indicating that Sesn-C may have acquired the mTORC1-regulating function by modifying the ancestral active site of an alkylhydroxyperoxidase during evolution. The C125S, H132A and Y127F mutations, which abrogate oxidoreductase activity of hSesn2 (Fig. 3d), did not disrupt the mTORC1-suppressing function of hSesn2 (Fig. 5b,d) and the DD motif mutations did not interfere with the oxidoreductase activity of hSesn2 (Fig. 3d). These results demonstrate that hSesn2 has two functional sites in separate domains (Sesn-A and Sesn-C), which independently regulate ROS levels and mTORC1 signalling. This unique feature makes the hSesn2 structure conceptually resemble the face of Janus—a twofold pseudo-symmetric molecule exhibiting two very distinct characteristics from its diametric domains (Fig. 6c).

### hSesn2 is not a structural homologue of human GDI1

hSesn2 was recently suggested to serve as a guanosine nucleotide dissociation inhibitor (GDI) for Rag GTPases^17^. This hypothesis was based on a very limited sequence homology between hSesn2 and human GDI1 (hGDI1)^17^. The overall structure of hGDI1 (PDB ID: 1UKV) is, however, very different from that of hSesn2 (Supplementary Fig. 9a). The putative GDI motif in hSesn2 also does not show any structural resemblance with the corresponding region in hGDI1 as shown in Supplementary Fig. 9b. Suggested key residues in the proposed GDI motif, such as Lys422 and Lys426 in hSesn2, are buried inside the structure, while another key residue Arg419 is surface exposed (Supplementary Fig. 9b,c). Mutation of these residues (R419A or R419A/K422A/K426A) did not abolish mTORC1-inhibiting function of hSesn2 (Fig. 5c), suggesting that the DD motif, not the putative GDI motif proposed by the former study^17^, constitutes the functional site responsible for mTORC1 inhibition.

### The DD motif is critical for hSesn2–GATOR2 association

According to the other recent reports^27^,^28^,^29^, hSesn2 controls mTORC1 activity through modulation of the GATOR1–GATOR2 complexes. The GATOR1 complex, which contains NPRL2, NPRL3 and DEPDC5, functions as a GTPase-activating protein (GAP) for Rag GTPase and thereby inhibits the localization of mTORC1 at lysosomes^30^. The GATOR2 complex, composed of WDR24, WDR59, SEC13, SEH1L and MIOS, blocks the GAP activity of GATOR1 and thereby potentiates mTORC1 activation^30^. hSesn2 directly binds to the GATOR2 complex and liberates GATOR1 from GATOR2-mediated inhibition, leading to mTORC1 inactivation^27^,^28^,^29^. Although we did not detect the direct interaction between hSesn2 and Rag GTPases (Supplementary Fig. 9d-f), we confirmed that hSesn2 exhibits a strong physical interaction with GATOR2 *in vivo* (Fig. 6d and Supplementary Fig. 9f) and *in vitro* (Fig. 6e), consistent with these studies^27^,^28^,^29^. Based on our structural and cell biological results, we reasoned that the helix–loop structure of Sesn-C might provide the surface for a protein–protein interaction particularly between hSesn2 and GATOR2. To test this idea, we examined the physical interactions between GATOR2 and hSesn2 variants, including WT, C125S, RDD and individual R404A, D406A and D407A mutants of hSesn2. Our co-immunoprecipitation analysis revealed that hSesn2-WT, as well as hSesn2-C125S and hSesn2-R404A, interact well with GATOR2; however, the RDD mutation, as well as individual D406A and D407A mutations, almost completely abolished the physical interaction between hSesn2 and GATOR2 (Fig. 6d,e and Supplementary Fig. 8b,c), suggesting that the DD motif in the helix–loop region of Sesn-C (Fig. 2b) are indeed critical for physical interaction with the GATOR2 complex and subsequent modulation of mTORC1 signalling (Fig. 6c).

## Discussion

Although the physiological significance of Sestrin proteins has been well-documented, the biochemical and functional understanding of this protein family has been very limited mostly due to the absence of knowledge about its structure. Here we present the crystal structure of full-length hSesn2, as the first molecular portrait for the Sestrin-family proteins. The crystal structure of hSesn2 reveals two structurally similar subdomains (Sesn-A and Sesn-C), whose biological functions have developed differently during evolution, with an antioxidant function and an mTORC1-inhibitory function, respectively.

Although a number of studies indicate that hSesn2 is an important cellular suppressor of ROS^2^,^6^,^13^,^15^,^16^,^31^,^32^,^33^,^34^, there has been tremendous controversy over whether hSesn2 indeed possesses oxidoreductase activity. This is because the originally proposed catalytic activity of hSesn2 as an ATP-dependent sulfinic acid reductase^2^ was not detectable in follow-up studies^35^,^36^. From our hSesn2 structure, we were also unable to detect any structural similarity between hSesn2 and sulfiredoxin^37^, the only known cysteine sulfinic acid reductase^38^,^39^. However, the crystal structure of hSesn2 revealed that both Sesn-A and Sesn-C domains of hSesn2 bear the structural resemblance with *R. eutropha* YP_296737.1, which belongs to a family of alkylhydroperoxidases including *M. tuberculosis* AhpD^21^. Sequence alignment suggested that Sesn-A and YP_296737.1, but not Sesn-C, possess a limited sequence similarity with the catalytic active site sequence of *M. tuberculosis* AhpD (Supplementary Fig. 5b), as initially reported^2^. The conserved regions in all three proteins exhibit the helix–turn–helix oxidoreductase motif^21^, although the relative position of the oxidoreductase motif within their primary sequence is different between AhpD and Sesn-A/YP_296737.1 (Supplementary Fig. 5a). The catalytic cysteine (Cys125) and residues in the proton delay system of AhpD-family oxidoreductases (Tyr127 and His132) are well-preserved within the Sesn-A domain^21^.

Based on the structural similarity with bacterial alkylhydroxyperoxidases, we predicted that Sesn-A may function as a direct scavenger for ROS. In particular, we reasoned that potential substrates of hSesn2 might be hydrophobic ROS because hydrophobic surface residues surround the active site of Sesn-A (Supplementary Fig. 7). Indeed, hSesn2 was able to efficiently eliminate bulky hydrophobic ROS (that is, cumene hydroperoxide), but not small hydrophilic ROS (that is, hydrogen peroxide) (Fig. 3). Mutations of the key catalytic residues in the helix–turn–helix oxidoreductase motif of Sesn-A (Fig. 2b), such as Cys125, Tyr127 and His132, abolished the catalytic activity of hSesn2 as an alkylhydroperoxidase (Fig. 3), indicating that this motif is indeed responsible for Sesn-A's catalytic activity. Considering that genetic loss of Sestrins can provoke diverse age- and obesity-associated pathologies such as type II diabetes, neurodegeneration and cardiovascular diseases^4^,^7^,^9^,^40^,^41^,^42^, one of the possible physiological substrates for hSesn2 is lipid peroxides, which are a family of hydrophobic alkylhydroperoxides known to promote the pathogenesis of these diseases^43^,^44^,^45^,^46^. The complete redox cycle of Sestrins, including its physiological substrates and reducing partners, awaits further investigation.

Although Sesn-C is structurally similar to Sesn-A and YP_296737.1, the helix–turn–helix oxidoreductase motif is not conserved in Sesn-C. Instead, Sesn-C has a helix–loop structure with the DD motif (Fig. 2b). Interestingly, the overall sequence of Sesn-C, including the helix–loop structure and other surface residues, is highly conserved across all Sestrin-family proteins (Supplementary Fig. 2a,b). Considering that the mTORC1-suppressing function of hSesn2 is independent of its redox-regulating function, we reasoned that Sesn-C might have acquired a new function as an mTORC1 inhibitor while having lost its ancient redox activity. To test this possibility, we introduced a series of mutations on the conserved surface of Sesn-C and looked for the mutant that abrogates its mTORC1-inhibitory function (Fig. 5a-c). Although most point mutations in the Sesn-C domain, including the formerly reported mutations in the putative GDI motif^17^, did not affect the mTORC1-inhibiting function of hSesn2 (Fig. 5b,c), the DD motif was critical for the mTORC1-inhibitory function of hSesn2. Because mutations of Cys125/Tyr127/His132 do not affect hSesn2's mTORC1-inhibiting function (Fig. 5b,c) and mutations of the DD motif do not affect hSesn2's oxidoreductase function (Fig. 3d), the function of Sesn-A and Sesn-C domains appears to be independent from each other. However, it should be further investigated whether the activities of the two domains exhibit any cross-talk in some cellular contexts. For example, the mTORC1-inhibiting and autophagy-activating functions of Sesn-C can alter the cellular redox status by indirectly promoting the activity of an antioxidant transcription factor Nrf2 (ref. 47). In addition, the role for Sesn-B domain also needs to be clarified. Because of its close proximity to the DD motif (Fig. 1a), Sesn-B may be involved in the regulation of the activity of Sesn-C in controlling mTORC1.

To explain how hSesn2 inhibits mTORC1, three competing mechanisms have been proposed in the field. (1) The first hypothesis is that hSesn2 inhibits mTORC1 by potentiating AMPK-mediated activation of TSC2, a Rheb-GAP^3^. Our data support this hypothesis by demonstrating that the DD motif in Sesn-C is required for the AMPK-activating function of hSesn2 (Fig. 6b). Although most literature consistently support that hSesn2 can provoke AMPK activation^4^,^8^,^27^,^40^,^48^,^49^,^50^, hSesn2 is still able to suppress mTORC1 in *AMPK*-null cells^27^,^28^,^29^, suggesting that there are additional pathways that connect hSesn2 with mTORC1 inhibition. (2) The second hypothesis is that hSesn2 functions as a GDI for Rag GTPases^17^. hSesn2 and Rags, however, do not seem to make a stable complex, as we were unable to detect a physical interaction between them either *in vitro* (Supplementary Fig. 9d.e) or *in vivo* (Supplementary Fig. 9f). The crystal structure of hSesn2 does not resemble that of any known GDIs (Supplementary Fig. 9a), and the putative GDI motif is in a different geometrical shape in the structure (Supplementary Fig. 9b). The putative GDI motif was also dispensable for hSesn2's mTORC1-inhibitory function (Fig. 5c). Although it is unlikely that hSesn2 can function as a stand-alone GDI, it is possible that hSesn2 can control Rag activities through additional proteins. (3) The third hypothesis is that hSesn2 indirectly regulates Rag GTPase through a physical interaction with GATOR2 (refs 27, 28, 29). GATOR2 is an inhibitor of GATOR1, a GAP for Rag GTPases. Direct association of hSesn2 with GATOR2 releases GATOR1 from the GATOR1–GATOR2 complexes, which triggers the GAP activity of GATOR1 towards Rag GTPases. In the current study, we have demonstrated that the DD motif of hSesn2 is critical for the physical association between hSesn2 and the GATOR2 complex (Fig. 6d,e and Supplementary Fig. 8b,c). Because the Rag GTPases serve as a metabolic switch that can reciprocally regulate AMPK and mTORC1 pathways^51^ and the GATOR complex is one of the critical regulators of this switch^52^,^53^, hSesn2-mediated inhibition of GATOR2 and subsequent activation of GATOR1 (refs 27, 28, 29) may be the key signalling event that affects both the AMPK and mTORC1 signalling pathways.

Through X-ray crystallography and structure-guided molecular biology experiments, we demonstrate the biochemical and structural basis of hSesn2's physiological functions. Janus-faced hSesn2 reduces alkylhydroperoxide radicals and suppresses mTORC1 signalling through its two independent functional motifs that face diametrically opposed directions (Fig. 6c). Considering that both chronic accumulation of ROS and prolonged activation of mTORC1 are well-known contributors to aging and age-associated diseases, these biochemical activities provide a highly convincing explanation for how Sestrin-family proteins function as a versatile anti-aging molecule^1^,^19^.

## Methods

### Protein expression and purification

The N-terminal 6 × histidines and maltose-binding protein (hisMBP)-tagged full-length hSesn2 was expressed in *Escherichia coli* Rosetta (DE3) with auto-inducible media^54^. Cells were harvested and resuspended in 30 mM Tris-HCl (pH 8.0), 500 mM NaCl and 5 mM β-mercaptoethanol with protease inhibitor cocktails. After sonication, soluble protein lysates were obtained from centrifugation at 20,000*g* for 30 min. The lysates were then applied into cobalt (Co) affinity chromatography (New England Biolabs) and eluted with the elution buffer (30 mM Tris-HCl (pH 8.0), 500 mM NaCl, 300 mM imidazole and 5 mM β-mercaptoethanol). The elution fraction was immediately dialysed against a buffer containing 30 mM Tris-HCl (pH 8.0), 100 mM NaCl and 5 mM β-mercaptoethanol with Tobacco Etch Virus protease (1:100 ratio) to cleave hisMBP-tag. The dialysed sample was re-applied into the Co-affinity chromatography to remove hisMBP-tag and the flow-through fraction was applied into HP Q chromatography (GE healthcare) pre-equilibrated with 30 mM Tris-HCl (pH 8.0), 100 mM NaCl and 1 mM DTT. hSesn2 was eluted by gradually increasing salt concentration up to 500 mM NaCl. Fractions that contain hSesn2 were pooled, concentrated and applied into the Superdex200 (10/300) size-exclusion chromatography (GE healthcare) pre-equilibrated with 30 mM Tris-HCl (pH 8.0), 100 mM NaCl and 1 mM TCEP (tris [2-carboxyethyl] phosphine). SeMet-substituted hSesn2 was expressed with PASM-5052 auto-inducible medium^54^. All wild-type and mutant proteins of hSesn2, as well as *M. tuberculosis* AhpD were cloned, expressed and purified in the same way. *M. tuberculosis* AhpD complementary DNA (MtCD00590021) was obtained from the DNASU plasmid repository and subcloned into the hisMBP vector. *M. tuberculosis* AhpC protein was kindly provided by Dr L.B. Poole (Wake Forest School of Medicine).

### Crystallization and data collection of hSesn2

Crystals of SeMet-substituted hSesn2 were grown using hanging drop vapour diffusion method at room temperature by mixing with a reservoir solution of 0.1 M MES (pH 6.5) and 1.15 M sodium malonate in a 1:1 ratio. Crystals were flash-frozen in liquid nitrogen after soaking in a cryo-protectant solution consisting of the well solution with 30% glycerol. SAD data (3.5 Å resolution) of the diamond-shaped crystals, in the space group I23, *a*=*b*=*c*=292.7 Å, *α*=*β*=*γ*=90°, were collected in LS-CAT beamline 21-ID-D (Advanced Photon Source, Argonne National Laboratory) at the peak wavelength of selenium.

### Data processing and structure determination of hSesn2

A total of seven SAD data sets from SeMet-labelled hSesn2 crystals were indexed, integrated, scaled and merged together by XDS^55^ through Xia2 (ref. 56). A total of 60 selenium sites from 5 copies of hSesn2 within the ASU were found using SHELXC/D/E^57^. For phasing, initial SAD phase was calculated by PHENIX.autosol^58^. Phases for both enantiomorphs were generated and the handedness was chosen by visual inspection of the electron density map. The phase was then subjected to automatic density modification with solvent flattening in the PHENIX programme RESOLVE^59^ to improve the phase. High solvent content (68%) and high copy number of hSesn2 within ASU enabled us to generate an electron density map whose quality is good enough to visualize all of the helices with mostly distinguishable side chains (Supplementary Fig. 1a). The α-helix-dominant structure and 12 SeMet positions within hSesn2 were helpful in assigning the sequence to the electron density map. The model was manually built using the programme COOT^60^ and refinements were carried out using REFMAC5 (ref. 61) with NCS restraints. The final refined model, except for residues 1–65, 221–224, 240–255, 272–279, 295–307, 329–332 and 479–480, contains *R*/*R*_free_=24.3/26.9.

### Size-exclusion chromatography with multi-angle light scattering

The average molecular weight (*M*_W_) and hydrodynamic radius (*R*_H_) of hSesn2 were determined by separation using a WTC-050S5 SEC column (Wyatt Technology Corp.) with an ÄKTAmicro (GE Healthcare) and by analysis with a DAWN HELEOS II MALS detector equipped with a WyattQELS dynamic light scattering detector and Optilab rEX differential refractive index detector, using ASTRA VI software (Wyatt Technology). The *M*_w_ was determined from the Raleigh ratio calculated by measuring the static light scattering and corresponding protein concentration of a selected peak. Bovine serum albumin served as a calibration standard. For size-exclusion chromatography with multi-angle light scattering (SEC-MALS), 60 μM hSesn2 were equilibrated in the running buffer (30 mM HEPES (pH 8.0), 100 mM NaCl and 1 mM TCEP) for 20 min on ice.

### Measurement of DTT oxidation

WT and mutant hSesn2 proteins, as well as *M. tuberculosis* AhpC and AhpD (positive control) and *E. coli* NemR^C106 only^ (negative control)^25^ proteins, purified as described above, were subjected to a DTT oxidation assay using cumene hydroperoxides. The rates of DTT oxidation catalysed by NemR^C106 only^, *M. tuberculosis* AhpC/AhpD, hSesn2-WT and hSesn2-mutants in the presence of cumene hydroperoxide were measured by monitoring the changes in absorbance at 310 nm due to formation of the DTT disulfide, using a Cary 50 spectrophotometer^62^. The proteins were initially reduced by 5 mM DTT, which was then removed by a desalting column (Thermo Scientific) before reaction. The assay was initiated by adding 15 mM of cumene hydroperoxide into a reaction solution containing 10–50 μM of purified proteins, 5 mM DTT, 1 mM EDTA and 100 mM KP_i_ (pH 7.0), and incubated in a 0.5 ml quartz cuvette at 25 °C. Each value was corrected for the background oxidation of DTT by cumene hydroperoxide in the absence of enzymes. The initial rate of DTT oxidation was obtained by calculating the slope over the first 5–10 s after the addition of cumene hydroperoxide and using the published extinction coefficient of oxidized DTT (110 M^−1^cm^−1^). Three different concentrations between 10 and 50 μM of enzymes were used for the assays, and the initial rates calculated from these experiments were linearly proportional to the enzyme concentration, enabling us to calculate *k*_cat_ values from these independent results.

### Measurement of peroxide reduction

The ferrous oxidation–xylenol orange (FOX) assay^63^,^64^ was used to determine the peroxidase activity of hSesn2-WT, hSesn2-C125S and *M. tuberculosis* AhpD. Working FOX reagent was freshly prepared by the addition of 1/100 volume of FOX reagent A (25 mM ammonium ferrous sulfate in 2.5 M sulfuric acid) into FOX reagent B (100 mM sorbitol and 125 mM xylenol orange). Before peroxidase assay, 10 or 25 μM of proteins were reduced by 0.1 mM or 0.25 mM DTT in a reaction solution (30 mM HEPES (pH 7.0), 100 mM NaCl) for hydrogen peroxides or cumene hydroperoxides, respectively. The reaction was initiated by adding 60 μM of hydrogen peroxides or 150 μM of cumene hydroperoxides into the protein-reaction solution mixture at room temperature. The concentrations of remaining peroxide were measured at various time points up to 50 min using the xylenol orange–iron reaction. The reactions were terminated at 50 min by mixing with 10 volumes of the working FOX reagent, and the mixtures were further incubated at room temperature for 30 min to allow colour development. The absorbance of the solution was measured at 560 nm. The peroxide concentrations were calculated by using a freshly prepared standard curve. All *P* values were calculated using the Student's *t*-test.

### Detection of cysteine sulfenic acids

To detect cysteine sulfenic acids of purified hSesn2, the proteins were treated with either reducing (1 mM DTT), oxidizing (120 μM cumene hydroperoxides) or blank agents for 30 min at 37 °C. Cysteine sulfenic acids were then labelled by 5 mM dimedone at 37 °C for 30 min, and detected by immunoblotting using a dimedone antibody (a kind gift from Dr K. Carroll)^65^. To detect cysteine sulfenic acids of endogenous hSesn2, RKO human colon cancer cells, cultured as described below, were treated with the indicated concentrations of cumene hydroperoxides. After thorough washing with PBS, cells were then lysed in a lysis buffer^4^ plus 5 mM dimedone for 30 min at room temperature. Clarified lysates were subjected to immunoprecipitation using either pre-immune IgG or hSesn2 antibodies as described below. Input (WCL) and immunopurified protein complex were analysed by anti-dimedone immunoblotting of a non-reducing SDS–PAGE gel. For quantification of endogenous hSesn2 sulfenylation, dimedone band intensity was quantified through densitometry, normalized to the total immunopurified hSesn2 levels and expressed as relative values compared with the untreated levels.

### Cell culture, transfection and lentiviral infection

RKO cells are from American Type Culture Collection (ATCC), and HEK293 cells (293A substrain) are from Thermo Fisher. Cells were cultured in Dulbecco's modified Eagle's medium (DMEM, Invitrogen) containing 10% foetal bovine serum and penicillin/streptomycin at 37 °C in 5% CO_2_. RKO cells were cultured in low-oxygen condition (5% O_2_) for *in vivo* sulfenylation assays. pLU-CMV–Flag–hSesn2-WT, pLU-CMV–Flag–hSesn2-C125S, pLU-CMV-GFP^2^, pRK7–HA–S6K1 (ref. 66), pRK5-GST–HA–Rags and pRK5–HA–GATOR2 (ref. 30) were used. Other hSesn2-mutants were generated by site-directed mutagenesis of wild-type hSesn2 complementary DNA and subcloned into the pLU-CMV–Flag plasmid. Lentiviruses were generated from the corresponding pLU constructs at the UM Vector Core. For exogenous protein expression, HEK293 cells were transfected with purified plasmid constructs and polyethylenimine (PEI, Sigma)^67^ or infected with replication-deficient lentiviral particles^68^. Cells were harvested 2 days after treatments for immunoblotting or immunoprecipitation experiments.

### Immunoprecipitation and immunoblotting

Cell and tissue lysates were prepared in a lysis buffer^4^ containing 0.3% CHAPS and protease inhibitor cocktail (Roche), and immunoprecipitated with anti-HA (A2095, Sigma) or anti-Flag (A2220, Sigma) agarose bead or hSesn2 antibody (8487, Cell Signaling, 1:100) conjugated to a protein G/A bead (Calbiochem). The beads were then washed four times with the lysis buffer. Whole cell lysates and the purified immunocomplexes were boiled in SDS sample buffer for 5 min, separated by SDS–PAGE, transferred to PVDF membranes and probed with indicated primary antibodies. HA (3F10, Roche, 1:1,000), Flag (M2, Sigma, 1:1,000), Actin (9E10, Developmental Studies Hybridoma Bank, 1:100), phospho-Thr389-S6K (9234, Cell Signaling, 1:1,000), pThr172-AMPK (2535, Cell Signaling, 1:1,000), pThr37/46 4E-BP (2855, Cell Signaling, 1:1,000), 4E-BP (9452, Cell Signaling, 1:1,000), phospho-Ser236/239-S6 (2211, Cell Signaling, 1:1,000), S6 (2317, Cell Signaling, 1:1,000), S6K (sc-230, Santa Cruz Biotechnology, 1:100), AMPK (sc-25792, Santa Cruz Biotechnology, 1:100) and hSesn2 (10795-1-AP, Proteintech, 1:1,000) antibodies were used for immunoblotting experiments. After incubation with secondary antibodies conjugated with horseradish peroxidase (Bio-Rad), chemiluminescence was detected using X-ray films or a LAS4000 (GE) system^69^. Uncropped images of immunoblots are provided in Supplementary Fig. 10.

### *In vitro* pull-down assay

HA–GATOR2 or Flag–hSesn2 (WT, C125S, D406A or D407A)-transfected HEK293 cell lysates were prepared in a lysis buffer containing 0.3% CHAPS and protease inhibitor cocktail, and immunopurified with anti-HA (A2095) or anti-Flag (A2220) agarose bead. The Flag–Sesn2 was then washed four times with the lysis buffer and eluted from the beads using Flag peptide (F3290, Sigma). For the pull-down assays, the anti-HA–GATOR2 immunocomplexes were washed four times with the lysis buffer and incubated overnight with purified Flag–hSesn2 proteins of WT, C125S, D406A or D407A at 4 °C. After extensive washing, the HA–GATOR2 complexes were eluted using HA peptide (I2149, Sigma). The pull-down eluates, as well as inputs were analysed through immunoblotting.

## Additional information

**Accession codes:** The coordinate and the structure factor for the reported crystal structure have been deposited with the Protein Data Bank under accession codes 5CUF.

**How to cite this article:** Kim, H. *et al.* Janus-faced Sestrin2 controls ROS and mTOR signalling through two separate functional domains. *Nat. Commun.* 6:10025 doi: 10.1038/ncomms10025 (2015).

## Supplementary Material

Supplementary Information Supplementary Figures 1-10, Supplementary Table 1 and Supplementary References

## Figures and Tables

**Table 1:** Data collection and refinement statistics for SAD (SeMet) structure.

	**hSesn2–SeMet**
*Data collection*	
Space group	I 2 3
Cell dimensions	
*a*, *b*, *c* (Å)	292.7
*α*, *β*, *γ* (°)	90
Resolution (Å)	44.1–3.5 (3.59–3.50)*
*R*_merge_	0.305 (2.201)
*R*_pim_	0.046 (0.335)
*I*/σ*I*	28.5 (3.7)
Completeness (%)	99.9 (100)
Redundancy	88.6 (86.2)
CC_1/2_ (%)	99.9 (87.8)
	
*Refinement*	
Resolution (Å)	44.1–3.5
No. of reflections	49798
*R*_work_/*R*_free_	0.243 / 0.269
No. of atoms	
Protein	15028
Ligand/ion	
Water	
*B*-factors	
Protein	94.7
Ligand/ion	
Water	
R.m.s deviations	
Bond lengths (Å)	0.0099
Bond angles (°)	1.5071
Ramachandran plot stats (%)	
Favoured	94.0
Allowed	6.0
Disallowed	0.0

SAD, single-wavelength anomalous diffraction.

^*^Highest resolution shell is shown in parenthesis.
