# Supplementary material for: Janus-faced Sestrin2 controls ROS and mTOR signalling through two separate functional domains
Source: Nat Commun. 2015 Nov 27;6:10025. doi: 10.1038/ncomms10025 (PMC4674687; doi:10.1038/ncomms10025)
Supplement: Supplementary Information — Supplementary Figures 1-10, Supplementary Table 1 and Supplementary References [file ncomms10025-s1.pdf]

a

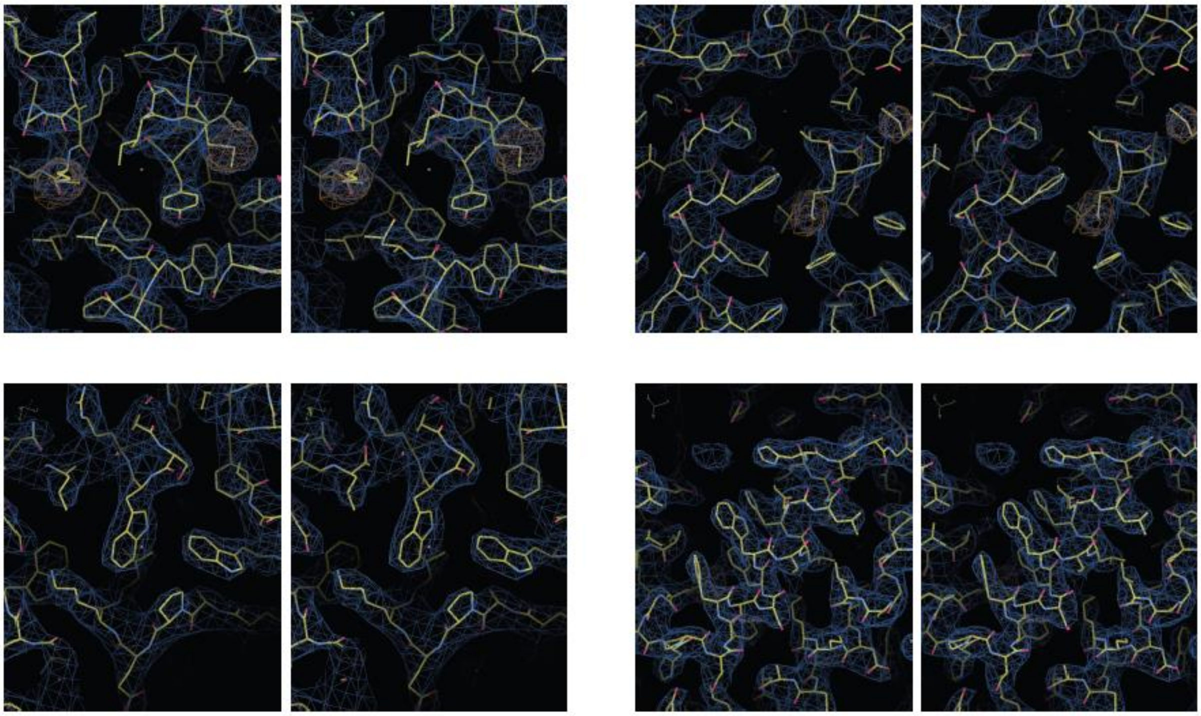

b

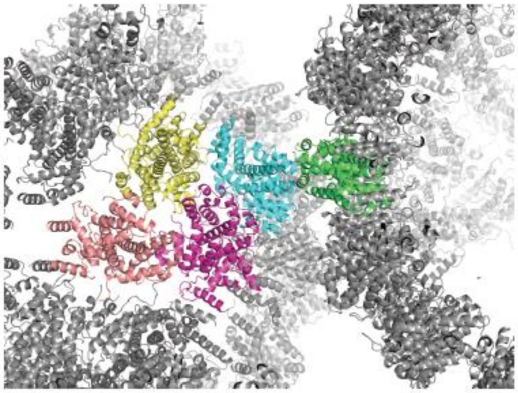

Supplementary Figure 1. Experimental electron density map with the stereo-view and the asymmetric unit of hSesn2. (a) Stereo images of the five-fold NCS-averaged experimental map of hSesn2 with 4.0 σ contour level at 3.5 Å resolution. Four representative map areas are magnified to visualize the overall quality of the map. The experimental map was calculated using the SAD method with SeMet-substituted hSesn2. Orange spheres represent the selenium sites generated by the anomalous difference map (10.0 σ contour level). The final model of hSesn2 is superimposed on the density map. **(b)** Five hSesn2 monomers within the asymmetric unit are displayed in yellow, orange, purple, cyan and green, whereas symmetry-related molecules surrounding the asymmetric unit are in grey.

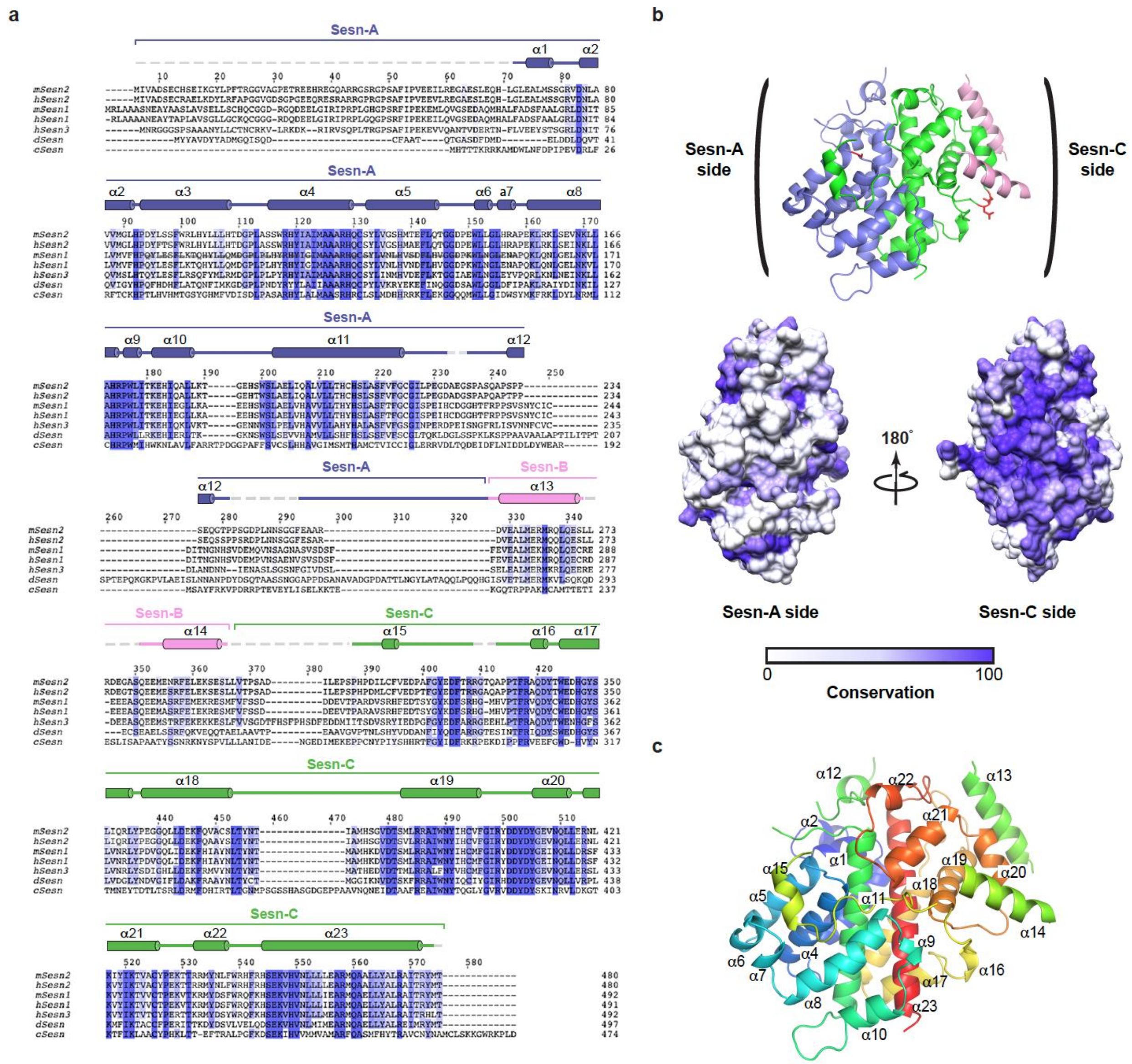

Supplementary Figure 2. Multiple sequence alignment of the Sestrin family. (a) Primary sequences of hSesn1 (GI 4092862), hSesn2 (GI 22900836), hSesn3 (GI 33878114), mouse Sestrin1 (GI 33585624), mouse Sestrin2 (GI 13542969), *Drosophila* Sestrin (GI 17862587), and *C. elegans* Sestrin (GI 373219509) were aligned using Clustal Omega[8]. The illustration for the alignment was generated with Jalview using default settings[9]. Highly conserved residues are shown in purple. The secondary structure identified from the hSesn2 crystal structure is shown above the sequence. Each domain is colored as in Fig. 1 and disordered regions are shown in grey dotted lines. **(b)** Surface residues of Sesn-C exhibit high levels of conservation across Sestrin family members. Colors of surface residues in Sesn-A side (left) and Sesn-C side (right) ramp from purple to white, corresponding to their degree of conservation in multiple sequence alignment analysis as shown in (a). **(c)** hSesn2 structure is shown in a ribbon diagram. Helices are labeled with numbers as shown in (a) and using rainbow color gradient from N-terminus (blue) to C-terminus (red).

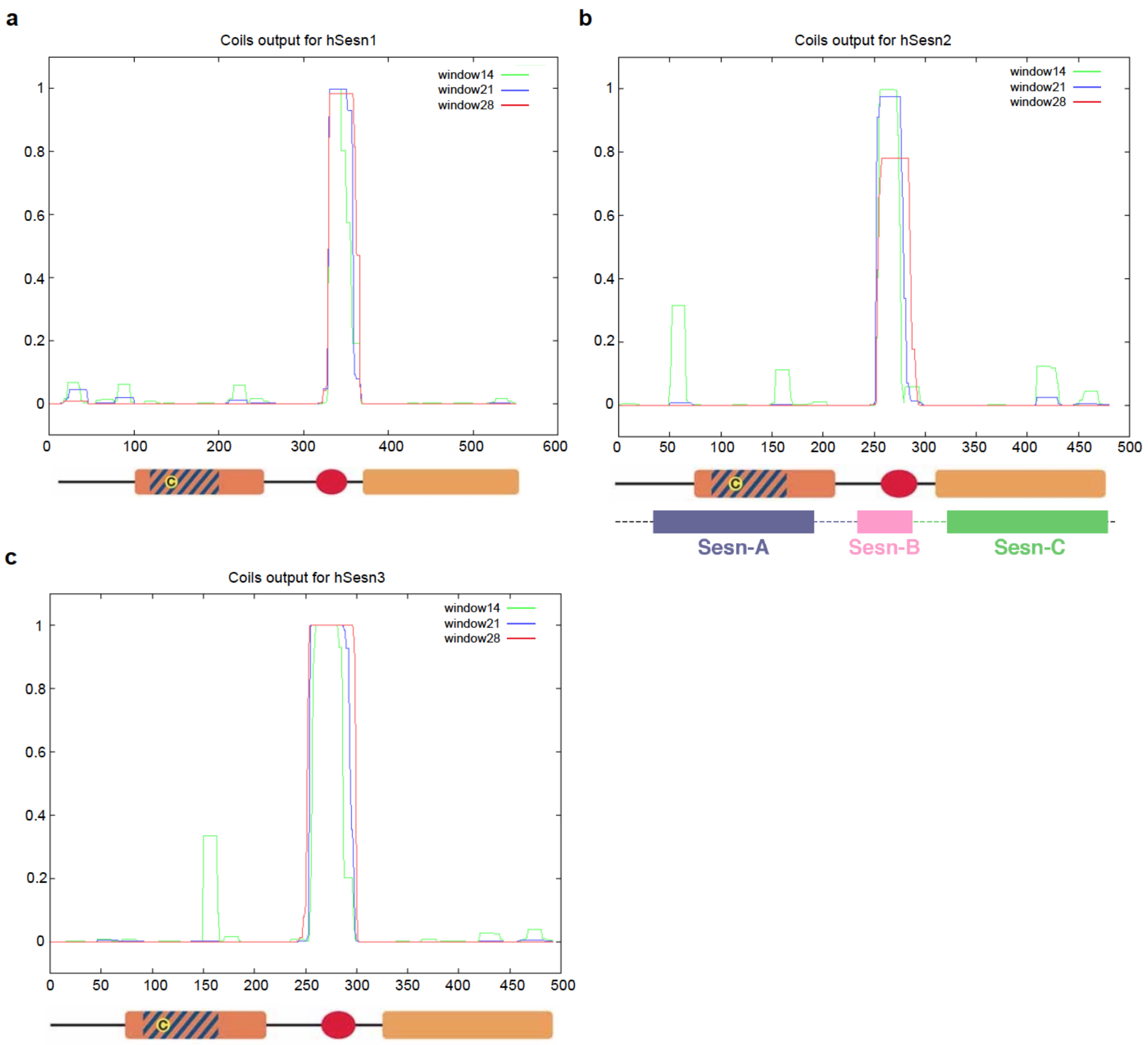

Supplementary Figure 3. Sesn-B constitutes a putative coiled-coil motif. (a-c) hSesn1-3 protein sequences were analyzed by the COILS program[10] to visualize the probability that a specific sequence region (in scanning windows of 14, 21 and 28 residues) contains a coiled-coil motif. However, COILS does not reach yes-or-no decisions based on a threshold value. Thus, even at high probabilities, there will be sequences that in fact do not form a coiled coil[10]. The domain structure illustration is according to a former study[11] as well as to the current X-ray crystal structure (**b**; see Fig. 1).

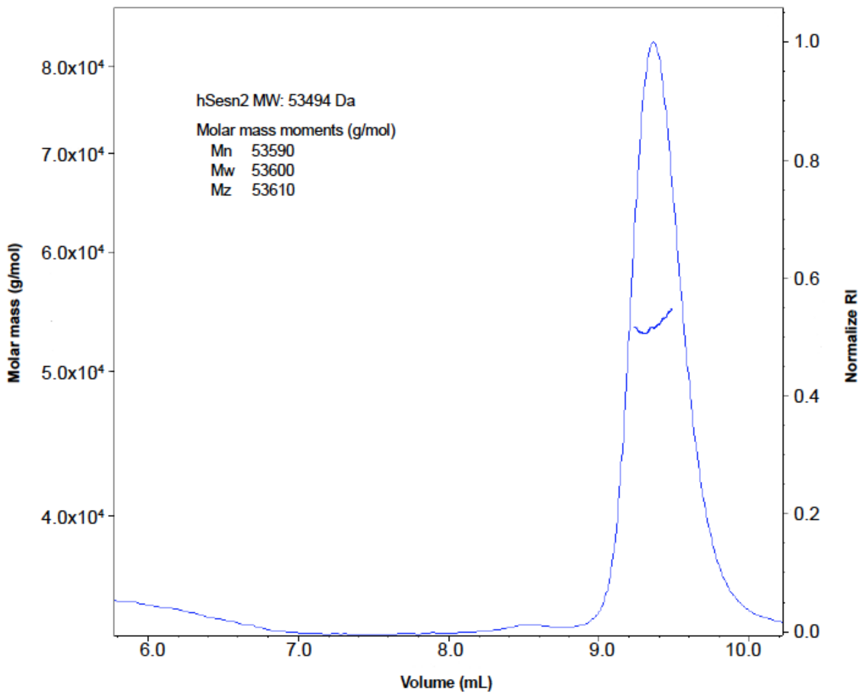

Supplementary Figure 4. hSesn2 exists as a monomer in solution. Size exclusion chromatography with multi-angle light scattering (SEC-MALS) profile of hSesn2 indicated that hSesn2 is predominantly monomeric in solution. The measured molecular weight of the single peak from the size exclusion chromatography (53,600 Da) is matched well with the molecular weight of hSesn2 (53,494 Da). Mw (Weight average molecular weight), Mn (Number average molecular weight), Mz (Higher average molecular weight), RI (refractive index).

a

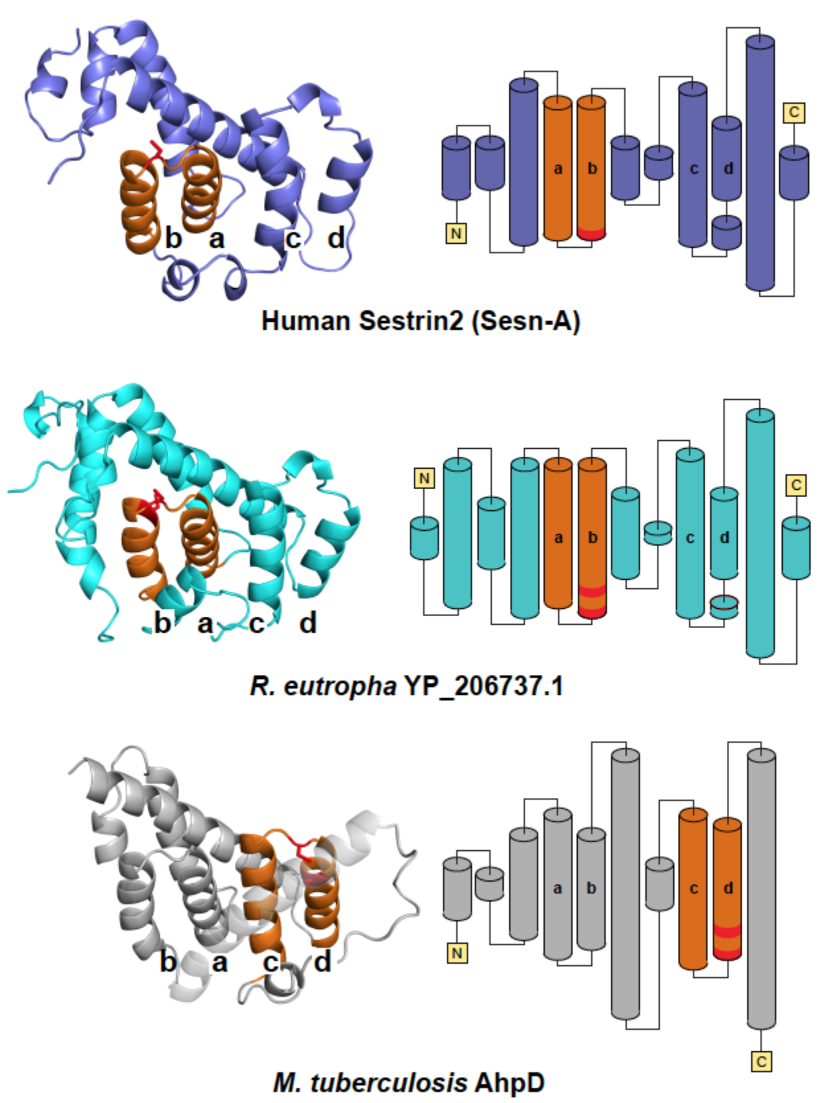

b

```
Mt_AhpD        --MSIEKLKAALPEYAKDIKLNLSSITRSSVLDQEQLWGTLLASAAATRNPQVLADIGAE   58
Sesn-A         MIVADSECRAELKDYLRF---APGGVGDSGP-GEEQ------RESRARRGPR-----GPS   45
YP_206737.1    ---------------------------------------------MTRPA-----HPI   8
                                                                 *

Mt_AhpD        ATMSIEKLKAALPEYAKDIKLNLSSITRSSVLDQEQ----LWGTLLASAAATRNPQVLAD   114
Sesn-A         AFIPVEEVLR-EGAESLEQHLGLEALMSSGRVDNLAVVMGLHPDYFTSFWRLHYLLLHTD   104
YP_206737.1    SRYPVPELAA-LPDD-IR-QRILEVQDKAGFVPNVFLTLAHRPDEFRAFFAYHDALMLKD   65
               :   : ::           :  *.    :. : :           : :    :   :  *

Mt_AhpD        IGAEATW----SFAVSAINGCSHCLVAHEHTLRTVGVDREAIFEALKAAAIVSGVA---Q   167
Sesn-A         GPLASSWRHYIAIMAAARHQCSYLVGSHMAEFLQTGGDPEWLLGL-------HRAPEKLR   157
YP_206737.1    GGLTKGEREMIVVATSAANQCLYCVVAHGAILRIYEKKPLVADQVAVN-YLKADIPPRQR   124
                           . .:* . * : : :*   :     .                     :

Mt_AhpD        ALA----------------TIEALSPS---------------------------------   178
Sesn-A         KLSEINKLLAHRPWLITKEHIQA---LLKTGEHTWSLAELIQALVLLTHCHSL-----SS   209
YP_206737.1    AMLDFALKVCKASHEVNEADFEALREHGFTDEDAWDIAAITAFFGLSNRMANTIGMRPND   184
                :                  ::*

Mt_AhpD        -------------------------------------------------   178
Sesn-A         FVFGCGILPEGDADGSPAPQAPTPPSEQSSPPSRDPLNNSGGFESAR      256
YP_206737.1    EFFLMGRVPKSK------------------------------------     196
```

Supplementary Fig. 5. Topology diagram comparison between human Sestrin2 domain A (Sesn-A), *R. eutropha* YP_296737.1 and *M. tuberculosis* AhpD. (a) 3D structure (left) and topology diagram (right) of hSesn2 Sesn-A domain, *Ralstonia eutropha* YP_206737.1 (PDB ID: 2PRR), and *Mycobacterium tuberculosis* AhpD (PDB ID: 1GU9). The four central helices are labeled from a to d, and the helix-turn-helix oxidoreductase motif, which contains the reactive Cys (colored in red), was colored in orange in both 3D structure and topology diagram. **(b)** Multiple sequence alignment between hSesn2, *R. eutropha* YP_206737.1, and *M. tuberculosis* AhpD. The conserved region (residues 109-139 of Sesn-A), which corresponds to the oxidoreductase motif of *M. tuberculosis* AhpD, is boxed in yellow color. The catalytic cysteine residues are highlighted in red.

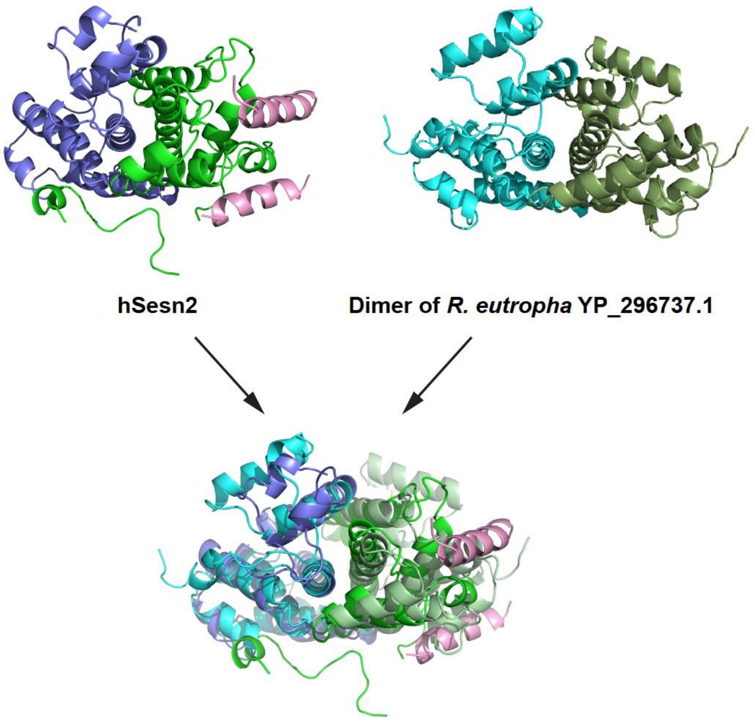

Supplementary Figure 6. Full-length hSesn2 monomer is structurally homologous to the *R. eutropha* YP_296737.1 dimer. Ribbon diagrams of the full-length hSesn2 monomer and the Dimer of *R. eutropha* YP_296737.1 are presented individually (upper panels) and in overlay (lower panel). Sesn-A, Sesn-B and Sesn-C domains in hSesn2 are colored in slate, pink and green, respectively. Two polypeptide chains in the Dimer of *R. eutropha* YP_296737.1 are colored in cyan (chain A) and pale green (chain B).

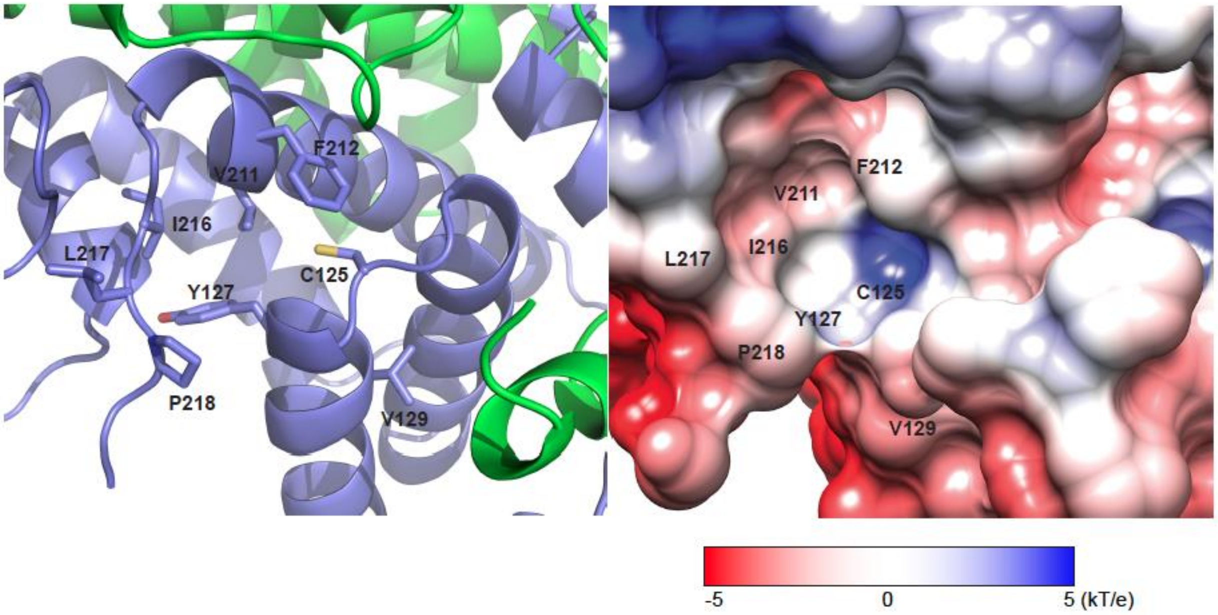

Supplementary Figure 7. Potential substrate access region near the active site of Sesn-A is surrounded by hydrophobic residues. Ribbon diagram (left) and electrostatic surface representation (right) of the putative active site for oxidoreductase activity of hSesn2, located in Sesn-A. The electrostatic potential was calculated by APBS[12]; positive potential is shown in blue and negative potential in red. Surface residues on the active-site area are labeled in both panels. These mostly hydrophobic residues generate a non-polar surface near the active site, which suggests that hSesn2 prefers hydrophobic substrates.

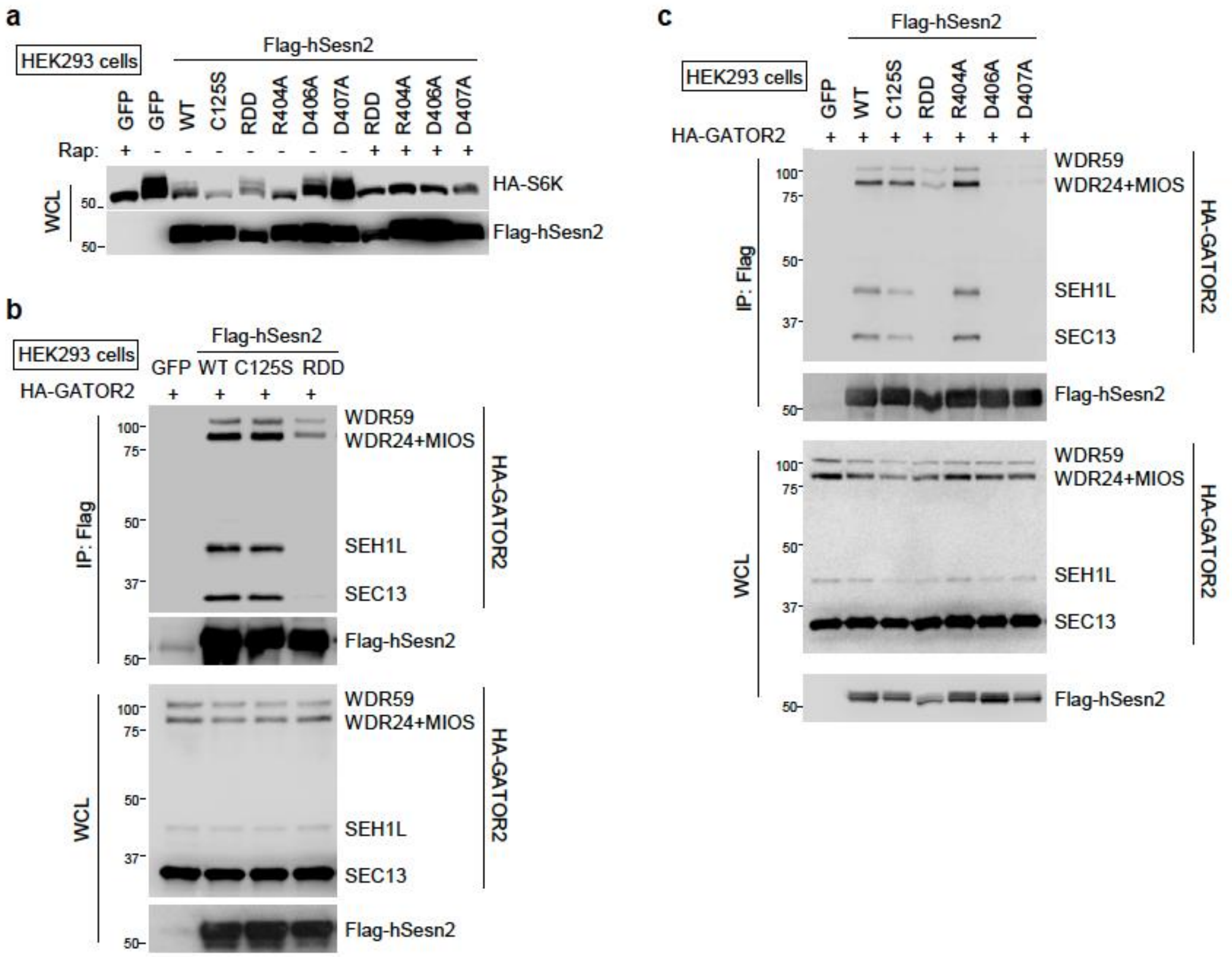

Supplementary Figure 8. Asp406 and Asp407 residues in the Sesn-C domain of hSesn2 are responsible for binding to the GATOR2 complex. (a) Asp406 and Asp407 are critical for the mTORC1-inhibiting activity of hSesn2. HEK293 cells were transfected with plasmid constructs expressing HA-tagged S6K1 and Flag-tagged hSesn2 of the indicated mutations. After 48 hr of transfection, cell lysates were analyzed by immunoblotting of the indicated proteins. GFP was used as a negative control. Rapamycin (Rap, 100 nM for 24 hr) was used as a positive control for mTORC1 inhibition. **(b)** Flag-tagged hSesn2 of indicated mutations were co-transfected with HA-tagged GATOR2 components (WDR59, WDR24, MIOS, SEH1L and SEC13) as indicated. Input (WCL) and Flag-immunopurified protein complex (IP) were analyzed by immunoblotting. **(c)** Asp406 and Asp407 in the active site of Sesn-C are critical for physical interaction between hSesn2 and GATOR2. Flag-tagged hSesn2 of the indicated mutations were co-transfected with HA-tagged GATOR2 components as indicated. hSesn2 was immunopurified using Flag antibody. Input (WCL) and immunopurified protein complex (IP) were analyzed by immunoblotting. RDD, R404A/D406A/D407A.

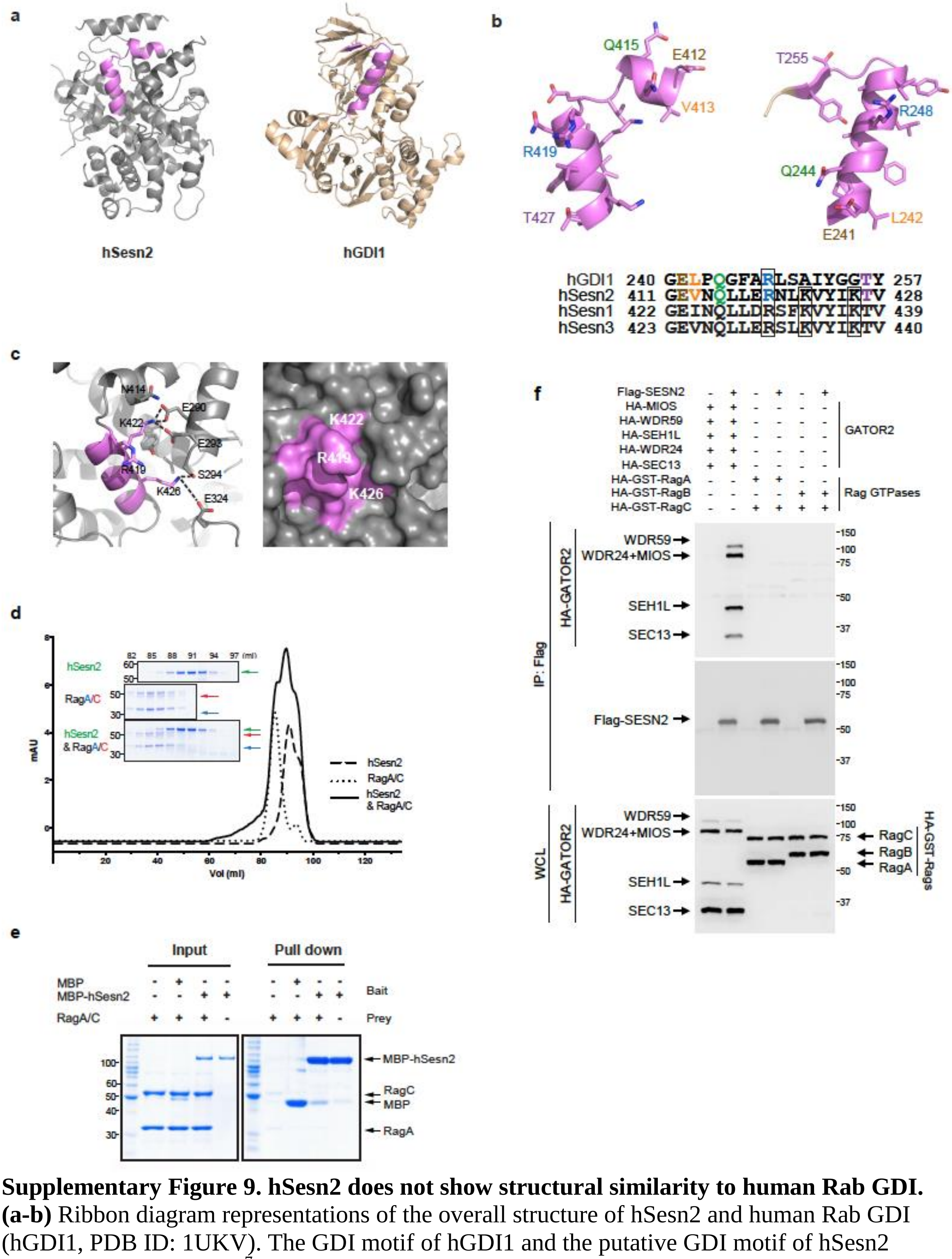

Supplementary Figure 9. hSesn2 does not show structural similarity to human Rab GDI. (a-b) Ribbon diagram representations of the overall structure of hSesn2 and human Rab GDI (hGDI1, PDB ID: 1UKV). The GDI motif of hGDI1 and the putative GDI motif of hSesn2 suggested by Peng *et. al.*[7] are highlighted in pink. **(b)** Magnification of the GDI motif of hGDI1 and the putative GDI motif of hSesn2. Corresponding amino acid sequences of hGDI1 and

hSesn1-3 are aligned and presented below the structure. Although there is limited sequence conservation between hSesn2 and hGDI1 at the primary amino acid sequence level (highlighted in the same colors), three-dimensional configurations of these residues are markedly different between the two structures. Residues that were suggested to be critical for GDI function of hSesn2 (Arg419, Lys422 and Lys426)[7], are boxed with solid lines in the sequence alignment. **(c)** The putative GDI motif of hSesn2 is displayed with a ribbon diagram (left) and space-filling representation (right). Lys422 and Lys426 are located in the second helix of the putative GDI motif (colored in pink). Because these lysine residues are highly conserved across Sestrin family members, they were suggested to have a potential catalytic function. However, the side chains of Lys422 and Lys426 do not have surface access and make multiple electrostatic interactions with neighboring residues. **(d)** hSesn2 does not associate with RagA/C GTPases in solution. Size-exclusion chromatography profiles of hSesn2 (dashed line), RagA/C heterodimer (dotted line), and hSesn2-RagA/C mixture (solid line). Coomassie blue staining images of peak fractions of each sample are shown as an inset. hSesn2 (green), RagA (blue) and RagC (red) protein bands are indicated by arrows of corresponding colors. The size-exclusion chromatography was pre-equilibrated with the buffer containing 30 mM Tris [pH 8.0], 100 mM NaCl, and 1 mM TCEP. **(e)** hSesn2 does not bind to RagA/C GTPases *in vitro* as indicated from pull down experiments using amylose resin and MBP-tagged hSesn2 as bait and RagA/C as prey. The result reveals no significant interaction between hSesn2 and RagA/C. The input and pull down results were visualized by coomassie blue staining of an SDS-PAGE gel. **(f)** hSesn2 does not interact with RagA/C or RagB/C GTPases in cells, although it strongly binds to the GATOR2 complex. GFP or Flag-tagged hSesn2 was co-transfected to HEK293 cells with HA-tagged GATOR2 components or Rag GTPases as indicated. hSesn2 was immunopurified using Flag antibody. Input (WCL) and immunopurified protein complex (IP) were analyzed by immunoblotting.

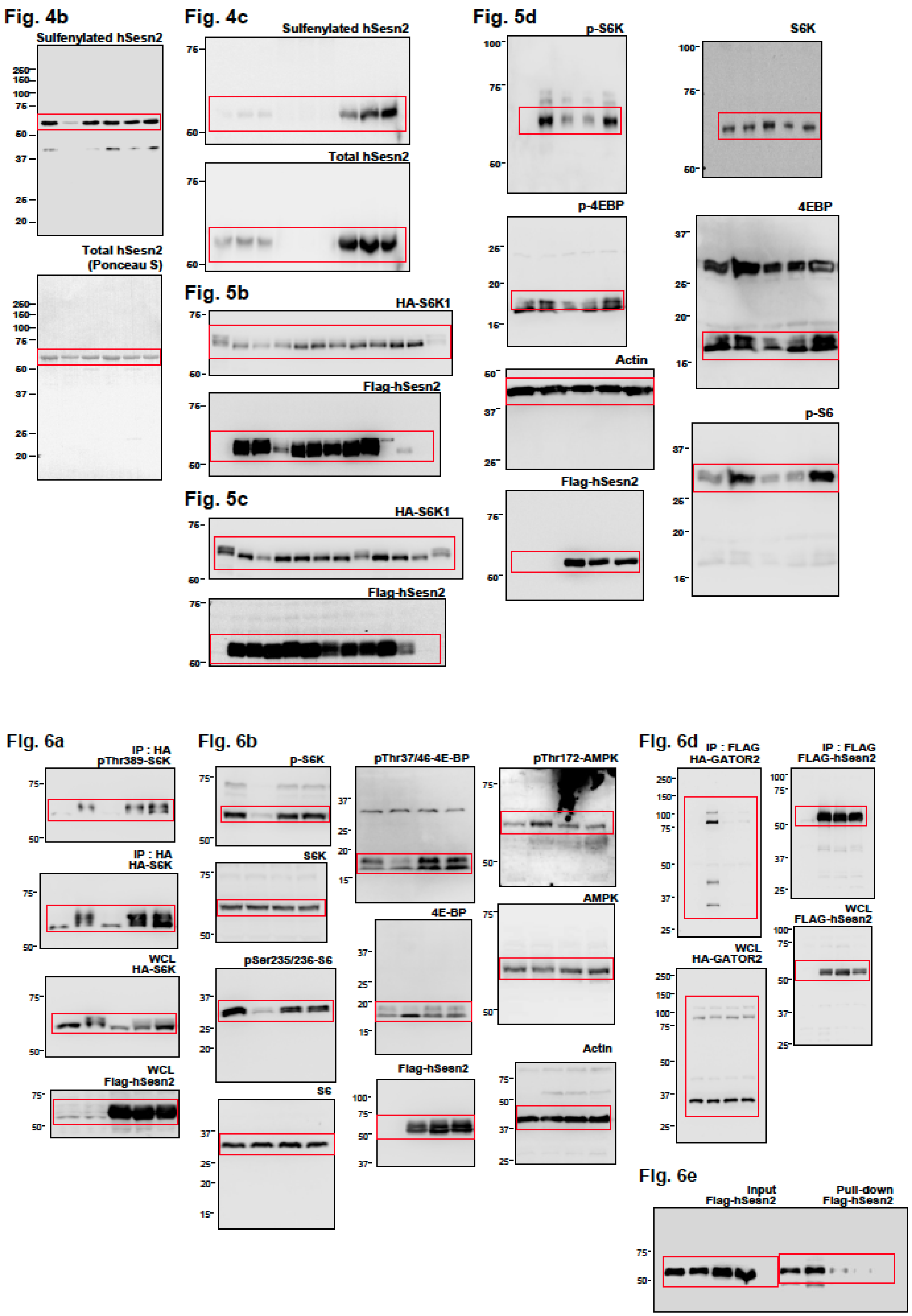

Supplementary Figure 10. Uncropped images of blots. Red boxes indicate the cropped regions. Molecular weight markers are indicated in kDa.

Supplementary Table 1. List of hSesn2 mutations used in this study.

Mutation	Rationale
Cys125Ser	This mutation disrupts the only remaining catalytic cysteine in Sesn-A. This mutation is formerly known to abolish hSesn2's antioxidant activity in cells and tissues[1, 2].
Leu128Cys/His113Glu	This mutation introduces the cysteine dyad in Sesn-A, and thus may boost hSesn2's antioxidant activity. However, this mutant was poorly expressed as a soluble protein in HEK293 cells or in *E. coli* (Fig. 5b and data not shown).
Pro87Ser	This variant was identified from a patient with myeloproliferative neoplasm[3].
His132Ala	Similar catalytic motif in *M. tuberculosis* AhpD[4-6] suggests that His132 is involved in the proton relay system while Tyr127 is more directly involved in the catalytic reduction process.
Tyr127Phe	
Cys204Ser	Cys204 in Sesn-A is the cysteine residue most closely located to Cys125. However, the distance between these cysteines is ~8 Å, which is not enough to form an intramolecular disulfide bond. In addition, Cys204 is not conserved in other Sestrins (hSesn1 and hSesn3) or in Sestrins from other species (mouse, *Drosophila* and *C. elegans*) (Supplementary Fig. 2a).
Cys214Ser	Cys214 is another cysteine in Sesn-A, conserved between hSesn1 and hSesn2 (Supplementary Fig. 2a).
Cys399Ser	These cysteine residues in Sesn-C were formerly suggested to partially mediate hSesn2's antioxidant function[1].
Cys430Ser	
Arg264Pro	These two mutations are expected to completely disrupt the putative coiled-coil motif present in Sesn-B (Supplementary Fig. 3).
Val258Arg/Glu259Leu/ Leu261Arg/Met262Arg/ Glu263Leu/Arg264Leu	
Thr336Ala/Phe337Ala	These five mutations represent the evolutionarily conserved surface regions in Sesn-C, which are subdivided into the five areas (Fig. 5a).
Gln340Ala/Asp341Ala	
Leu373Ala/Asn376Ala/ Asp385Ala/Ser387Ala	
Arg404Ala/Asp406Ala/ Asp407Ala	
Asp409Ala/Gly411Ala/ Gln415Ala	
Arg419Ala	These mutations, described by Peng *et al.*[7], were suggested to disrupt the putative GDI motif of hSesn2 (Supplementary Fig. 9b).
Arg419Ala/Lys422Ala/ Lys426Ala	

Supplementary References

1 Budanov, A. V., Sablina, A. A., Feinstein, E., Koonin, E. V. & Chumakov, P. M. Regeneration of peroxiredoxins by p53-regulated sestrins, homologs of bacterial AhpD. *Science* **304**, 596-600, (2004).

2 Ro, S. H. *et al.* Sestrin2 inhibits uncoupling protein 1 expression through suppressing reactive oxygen species. *Proc. Natl. Acad. Sci. USA* **111**, 7849-7854, (2014).

3 Hou, Y. *et al.* Single-cell exome sequencing and monoclonal evolution of a JAK2-negative myeloproliferative neoplasm. *Cell* **148**, 873-885, (2012).

4 Bryk, R., Lima, C. D., Erdjument-Bromage, H., Tempst, P. & Nathan, C. Metabolic enzymes of mycobacteria linked to antioxidant defense by a thioredoxin-like protein. *Science* **295**, 1073-1077, (2002).

5 Koshkin, A., Nunn, C. M., Djordjevic, S. & Ortiz de Montellano, P. R. The mechanism of Mycobacterium tuberculosis alkylhydroperoxidase AhpD as defined by mutagenesis, crystallography, and kinetics. *J. Biol. Chem.* **278**, 29502-29508, (2003).

6 Nunn, C. M., Djordjevic, S., Hillas, P. J., Nishida, C. R. & Ortiz de Montellano, P. R. The crystal structure of Mycobacterium tuberculosis alkylhydroperoxidase AhpD, a potential target for antitubercular drug design. *J. Biol. Chem.* **277**, 20033-20040, (2002).

7 Peng, M., Yin, N. & Li, M. O. Sestrins Function as Guanine Nucleotide Dissociation Inhibitors for Rag GTPases to Control mTORC1 Signaling. *Cell* **159**, 122-133, (2014).

8 Sievers, F. *et al.* Fast, scalable generation of high-quality protein multiple sequence alignments using Clustal Omega. *Mol. Syst. Biol.* **7**, 539, (2011).

9 Waterhouse, A. M., Procter, J. B., Martin, D. M., Clamp, M. & Barton, G. J. Jalview Version 2--a multiple sequence alignment editor and analysis workbench. *Bioinformatics* **25**, 1189-1191, (2009).

10 Lupas, A., Van Dyke, M. & Stock, J. Predicting coiled coils from protein sequences. *Science* **252**, 1162-1164, (1991).

11 Budanov, A. V., Lee, J. H. & Karin, M. Stressin' Sestrins take an aging fight. *EMBO Mol. Med.* **2**, 388-400, (2010).

12 Baker, N. A., Sept, D., Joseph, S., Holst, M. J. & McCammon, J. A. Electrostatics of nanosystems: application to microtubules and the ribosome. *Proc. Natl. Acad. Sci. USA* **98**, 10037-10041, (2001).
